# Structure–Activity Predictions From Computational Mining of Protein Databases to Assist Modular Design of Antimicrobial Peptides

**DOI:** 10.3389/fmicb.2022.812903

**Published:** 2022-04-15

**Authors:** Claudia Feurstein, Vera Meyer, Sascha Jung

**Affiliations:** Chair of Applied and Molecular Microbiology, Institute of Biotechnology, Technische Universität Berlin, Berlin, Germany

**Keywords:** antimicrobial peptide, antifungal peptide, data mining, data analysis, peptide database, AMP, gamma-core motif

## Abstract

Antimicrobial peptides (AMPs) are naturally produced by pro- and eukaryotes and are promising alternatives to antibiotics to fight multidrug-resistant microorganisms. However, despite thousands of AMP entries in respective databases, predictions about their structure–activity relationships are still limited. Similarly, common or dissimilar properties of AMPs that have evolved in different taxonomic groups are nearly unknown. We leveraged data entries for 10,987 peptides currently listed in the three antimicrobial peptide databases APD, DRAMP and DBAASP to aid structure–activity predictions. However, this number reduced to 3,828 AMPs that we could use for computational analyses, due to our stringent quality control criteria. The analysis uncovered a strong bias towards AMPs isolated from amphibians (1,391), whereas only 35 AMPs originate from fungi (0.9%), hindering evolutionary analyses on the origin and phylogenetic relationship of AMPs. The majority (62%) of the 3,828 AMPs consists of less than 40 amino acids but with a molecular weight higher than 2.5 kDa, has a net positive charge and shares a hydrophobic character. They are enriched in glycine, lysine and cysteine but are depleted in glutamate, aspartate and methionine when compared with a peptide set of the same size randomly selected from the UniProt database. The AMPs that deviate from this pattern (38%) can be found in different taxonomic groups, in particular in Gram-negative bacteria. Remarkably, the γ-core motif claimed so far as a unifying structural signature in cysteine-stabilised AMPs is absent in nearly 90% of the peptides, questioning its relevance as a prerequisite for antimicrobial activity. The disclosure of AMPs pattern and their variation in producing organism groups extends our knowledge of the structural diversity of AMPs and will assist future peptide screens in unexplored microorganisms. Structural design of peptide antibiotic drugs will benefit using natural AMPs as lead compounds. However, a reliable and statistically balanced database is missing which leads to a large knowledge gap in the AMP field. Thus, thorough evaluation of the available data, mitigation of biases and standardised experimental setups need to be implemented to leverage the full potential of AMPs for drug development programmes in the clinics and agriculture.

## Introduction

A well-established approach for the development of medical or agricultural antimicrobials is the use of natural peptides as scaffold to design novel synthetic derivatives. These peptides can be both of ribosomal and non-ribosomal origin and are found in pro- and eukaryotes as part of the first line defence against invading microorganisms and have various structural features (Fleming, 1929; Yeaman and Yount, 2007; Richter et al., 2014). The review of Koo and Seo (2019) lists 36 peptide antibiotics. Twenty-seven of them are in clinical phases I to III and nine are preclinical. Among them are also AMPs of ribosomal origin, as pexiganan (phase III), omiganan (phase III) and arenicin (preclinical). So far, the only FDA (food and drug association) approved peptide antibiotics are either of non-ribosomal origin or synthetic. The statement in the review by Zhang et al. (2021), where human lactoferrin peptide 1–11 (hLF1-11) is listed as being approved by the FDA could not be verified in our literature search. However, among the FDA approved synthetic peptides, there are a few representatives which could also be synthesised ribosomally and do not show specific modifications or special amino acid derivatives. This displays that AMPs (of ribosomal origin) are generally appropriate to reach late clinical trials and become approved. Hence, it might be only a matter of time but also of extended AMP basic research before ribosomally derived AMPs or their derivatives will pass phase III clinical studies, finally.

Generally, non-ribosomal antimicrobial peptides, like penicillin or vancomycin, are small, and their structures can be linear, cyclic or branched (Walsh, 2004; Tajbakhsh et al., 2017). Their characteristics include but are not limited to the usage of non-proteinogenic and D-amino acids, and posttranslational modification such as glycosylation, acetylation and methylation (Tajbakhsh et al., 2017). A paradigm for a non-ribosomal antimicrobial peptide is penicillin, which was discovered by Alexander Fleming in 1928 (Fleming, 1929). For many years, it was the lead compound to treat bacterial infections (Aminov, 2010). However, due to extended drug use, resistance mechanisms evolved in bacteria (Miller, 2002). Semisynthetic derivatives of penicillin were developed by chemical modification while maintaining the penicillin’s core region, a β-lactam thiazolidine ring system (Miller, 2002; Rolinson and Geddes, 2007). Nevertheless, bacteria rapidly evolved resistance to those derivatives as well. The history of penicillin explains the steady increase in multi-resistant bacteria and the decrease of available antibiotics.

Ribosomal antimicrobial peptides (AMPs), like AFP from *Aspergillus giganteus* or the human cathelicidin LL-37, are generally defined by a mean size of 20–40 amino acids, a net positive charge, and an amphipathic character (Yeaman and Yount, 2007; Bondaryk et al., 2017; Huan et al., 2020). Further classification of AMPs can be made regarding the accumulation of certain amino acid residues in their primary structure, e.g. glycine, proline, arginine, tryptophan or histidine, which can determine the AMPs’ modes of action (Huan et al., 2020). AMPs can also be classified based on their secondary structures which can include α-helices, β-sheets, linear extension or both α-helices and β-sheets (Reddy et al., 2004; Huan et al., 2020). Furthermore, primary- and secondary-structural features can form structural and/or functional motifs, for example the γ-core motif.

The γ-core motif was postulated as a unifying structural signature present in all cysteine-stabilised AMPs (Yount and Yeaman, 2004). AFP from *A. giganteus*, PAF from *P. chrysogenum*, HNP-3 from *H. sapiens* and Drosomycin from *D. melagonaster* are some examples for γ-core AMPs. It is a three-dimensional structural component in disulphide-stabilised AMPs, which has a positive net charge and an amphipathic character (Yount and Yeaman, 2004). This motif consists of two beta strands and a specific primary structure of one glycine and two cysteines appearing in three consensus pattern isoforms named dextromeric or D-isoform (NH_2_…[X_1–3_]-[GXC]-[X_3–9_]-[C]…COOH), levomeric 1 or L1-isoform (NH_2_…[C]-[X_3–9_]-[CXG]-[X_1–3_]…COOH) and levomeric 2 or L2-isoform (NH_2_…[C]-[X_3–9_]-[GXC]-[X_1–3_]…COOH; Yount and Yeaman, 2004; Yeaman and Yount, 2007). The γ-core motif was recently described to be an important structural component upon peptide-membrane interaction of AMPs due to its net positive charge and amphipathic surface properties (Yeaman and Yount, 2007; Tajbakhsh et al., 2017; Utesch et al., 2018).

Most structure–activity studies of AMPs focus on understanding the interaction of AMPs with molecular components of the target organisms of interest, including cell wall and plasma membrane, membrane and cytoplasmatic proteins, lipid biosynthetic pathways as well as DNA and RNA (Reddy et al., 2004; Gogoladze et al., 2014; Tajbakhsh et al., 2017). However, the correlation between an AMP structure and its target organism, potentially determining specificity towards target organisms, appears to be neglected. We hypothesise that AMP producing hosts have developed specific patterns for their AMPs to interact with surrounding microorganisms. Thereby, AMPs can either display a broad-spectrum activity or are specifically active against selected target organisms supposedly present in the same natural niche as the producing organism.

Broad-spectrum AMPs, such as human cathelicidin LL-37 (a pore-forming peptide) or pig protegrin-1, are not only active against microorganisms but also against cancer cells and can exert immunoregulatory functions (Scheenstra et al., 2019). LL-37 adopts an alpha-helical structure and has a net positive charge (+6) (Scheenstra et al., 2019). In contrast, the antifungal peptide AFP produced by the filamentous fungus *Aspergillus giganteus* is specifically active against various filamentous fungi, whereas yeast or bacteria, plant or mammalian cells are not affected (Meyer, 2008). AFP is the founder molecule of the AFP family of peptides which are small, amphipathic and cationic, adopt a beta-barrel structure consisting of five beta strands and which contain a γ-core motif (Meyer, 2008; Meyer and Jung, 2018; Paege et al., 2019). It interacts with lipids of the plasma membrane but also binds to the cell wall compound chitin (Hagen et al., 2007; Utesch et al., 2018). Furthermore, it was shown to inhibit the enzymatic activity of chitin synthases (Hagen et al., 2007). In contrast to LL-37, pore formation or additional functions of AFP, e.g. immunoregulation, are not reported. Both peptides, LL-37 and AFP, have different structural properties which exert different biological activities. Since this was only one example among the variety of AMPs, we aimed to identify basic structural properties of AMPs relevant for their antimicrobial activities. Hence, the three databases, Antimicrobial Peptide Database (APD), Data Repository of Antimicrobial Peptides (DRAMP) and Database of Antimicrobial Activity and Structure of Peptides (DBAASP), were mined to investigate three questions: (i) Do the structural properties of an AMP depend on its producing organism? (ii) Does the choice of the target organisms tested depend on the AMP producing organism? (iii) Does the activity of an AMP against a taxonomic group correlates with its structural properties? To address the first question, we specified 13 taxonomic groups known to produce AMPs, i.e. Gram-positive bacteria, Gram-negative bacteria, fungi, plants, arachnids, insects, crustaceans, molluscs, fish, amphibians, mammals (including AMPs of human origin), birds and reptiles. For the second and third question, we focused on filamentous fungi, yeast, Gram-positive bacteria, Gram-negative bacteria, virus, mammalian cells and mammalian cancer cells as target organisms.

## Materials and Methods

### AMP Database Mining and Data Processing

Data mining was performed on the three publicly accessible online platforms APD, DRAMP and DBAASP (Supplementary Additional Files 1–4; Gogoladze et al., 2014; Wang et al., 2016; Kang et al., 2019). Resulting peptide lists included website ID, peptide sequence, sequence length, peptide name, producer organism, target organisms, references and if available gene and PDB ID. After combining the data, further processing involved excluding peptides which (i) were listed twice based on the amino acid sequence (3,856 entries), (ii) synthetic (788 entries), (iii) containing non-canonical amino acids (283 entries), (iv) are not published in a peer-reviewed publication (282 entries) or (v) did not have a producer organism recorded (32 entries). AMPs showing identical amino acid sequences were further distinguished if they had a modification on the C- and/or N-terminus. In total, 161 peptides were modified at their termini, leading to 201 modified peptides. Highest modification rate was five per sequence.

Producer organisms were grouped in archaea, gram-positive and negative bacteria, fungi, plants, arachnids, insects, crustaceans, molluscs, fish, amphibians, mammals (including AMPs of human origin), birds, reptiles and other using the NCBI taxonomy browser (Schoch et al., 2020). Target organisms were assigned into the groups: filamentous fungi, yeasts, Gram-positive and negative bacteria, viruses, mammal cancer and non-cancer cells as well as other. The producer and target groups ‘other’ include organisms that could not be allocated to the other groups. Multiple AMPs of the three databases contained overlapping entries. Thus, if an organism on species level was tested more than once per peptide it was only counted once to avoid double count. The final processing step excluded peptides without a target given, the categories ‘other’ of both producer and target organisms and the peptides of the producer group archaea, since the number of peptides in those groups were not statistically evaluable. All data were processed according to their appearance on the databases and have not been inspected regarding completeness and correctness. A more detailed description of the data mining and processing procedure is available in Supplementary Additional File 1. The final list of AMPs and their primary structure characteristics is available in Supplementary Additional Files 5 and 6, respectively. Date of last access: October 2020.

All three databases were investigated for new entries in September 2021. APD showed 23 new entries, DBAASP showed 76 new entries and DRAMP showed 633 new entries. After automated application of exclusion criteria in the first round, solely 63 entries from DBAASP remained. This number of entries is expected to decrease further when additional manual editing of data is performed. In conclusion, the number of evaluable AMPs increased for merely 2% or even less within the period of 1 year which emphasises the validity of this *in silico* study.

### UniProt Data Mining and Processing

To put the statistical findings of the producer-structure relation in context, additional data were retrieved from UniProt (The UniProt Consortium, 2019). On the website reviewed, peptides of the Protein knowledgebase (UniProtKB) were retrieved using the taxonomy view. Here, taxonomy groups belonging to the AMP producer groups were chosen, peptides longer than 190 amino acids excluded, since this corresponds to the longest peptide of the analysed AMPs. Random peptides were chosen (i) in the same amount as the test producer organism groups and (ii) for each producer group 250 peptides. Afterwards, the corresponding peptides were analysed regarding their structural characteristics. A more detailed description of the data mining and processing procedure is available in Supplementary Additional File 2. The final list of UniProt peptides and their structural characteristics is available in Supplementary Additional Files 7–9.

### Data Analysis

Peptide evaluation included the following characteristics: charge at physiological pH (pH 7.4), the molecular weight, the length, the amino acid composition, the hydropathy (GRAVY: grand average of hydropathy, Scale as described in Eisenberg et al., 1984, column ‘Normalized consensus’) and the γ-core motif (Yount and Yeaman, 2004). The checked postulated characteristics of the γ-core motif include its primary structure, a positive charge at pH 7.4, and if it is hydrophobic (Yount and Yeaman, 2004). Those properties were correlated with the producer group and specific target organisms. Latter organisms include the filamentous fungi *Aspergillus* spp. and *Fusarium* spp., the yeasts *Candida* spp. and *Cryptococcus* spp. Additionally, the ESKAPE organisms (which is an acronym formed by the first letter of the six following genera) were examined, including the Gram-positive bacteria *Enterococcus faecium* and *Staphylococcus aureus* and Gram-negative bacteria *Klebsiella pneumoniae*, *Acinetobacter baumannii*, *Pseudomonas aeruginosa* and *Enterobacter* spp. Given, AMP concentrations were not evaluated, due to large variations regarding cultivation conditions and the reporting of the inhibiting concentration. Additionally, producer organism and target organism groups have been correlated by calculating (i) the mean of species tested per peptide of each target group, (ii) the mean of species tested per peptide and producer organism group of each target group and (iii) the maximum and minimum of species tested per producer organism group of each target group. Data were statistically assessed applying the Χ^2^ test [chi (capital Greek letter) square] of independence and the effect size measurement Cohen’s *ω* to generate the contingency table (Cohen, 1988; McHugh, 2013). The effect size according to Cohen can be small (0.1–0.29), medium (0.3–0.49) and large (≥0.5). Statistical evaluation could not have been applied if in the Χ^2^ test for more than 20% of relations tested the expected sample size was less than 5 (Cohen, 1988; McHugh, 2013). To normalise differences to the peptide amount of each producer group or target organism, percentages were used to generate heat maps. Non-ribosomal and ribosomal peptides were not distinguished since this information was only given by one of three databases.

**Table 1:** Maximum and minimum values of AMP properties in association with the producing organism group.

								Amino acid	
			Molecular weight	Charge pH 7.4	Length	GRAVY	γ-core motif		
								Highest	Lowest
Producer organism groups	Bacteria	Gram-positive	2,500 < MW	Positive	20 < L ≤ 40	Hydrophobic	L1	G/A/K	M/H/E
		Gram-negative	MW ≤ 2,500	Negative	L ≤ 20	Hydrophobic	D	G/A/S	H/M/W
		Fungi	2,500 < MW	Positive	20 < L ≤ 40	Hydrophilic	L2	G/C/K	M/W/Q
		Plants	2,500 < MW	Positive	20 < L ≤ 40	Hydrophilic	D/L2	C/G/R	M/W/H
	Invertebrates	Arachnids	2,500 < MW	Positive	20 < L ≤ 40	Hydrophobic	L2	G/K/L	M/W/H
		Insects	2,500 < MW	Positive	L ≤ 20	Hydrophobic	L2	G/K/A	M/W/Y
		Crustaceans	2,500 < MW	Highly positive	60 < L	Hydrophilic	D	G/P/R	M/W/H
		Molluscs	2,500 < MW	Positive	20 < L ≤ 40	Hydrophilic	L1	C/G/R	M/W/H
	Vertebrates	Fish	2,500 < MW	Positive	20 < L ≤ 40	Hydrophobic	D	G/K/R	M/W/Y
		Amphibians	MW ≤ 2,500	Positive	20 < L ≤ 40	Hydrophobic	L2	L/K/G	Y/W/H
		Mammals	2,500 < MW	Highly positive	20 < L ≤ 40	Hydrophilic	L2	R/K/G	M/W/H
		Birds	2,500 < MW	Highly positive	20 < L ≤ 40	Hydrophilic	D	R/C/G	M/E/D
		Reptiles	2,500 < MW	Highly positive	20 < L ≤ 40	Hydrophilic	D	K/R/G	M/W/H
		Total	2,500 < MW	Positive	20 < L ≤ 40	Hydrophobic	L2	G/K/L	H/M/W
		*p*-value (total)	<0.01	<0.01	<0.01	<0.01		<0.01	
		Cohen’s ω (total)	0.30	0.57	0.48	0.58		0.35	

## Results

### The Molecular Patterns of AMPs Are Associated With the Producing Organism Groups

**Figure 1:**
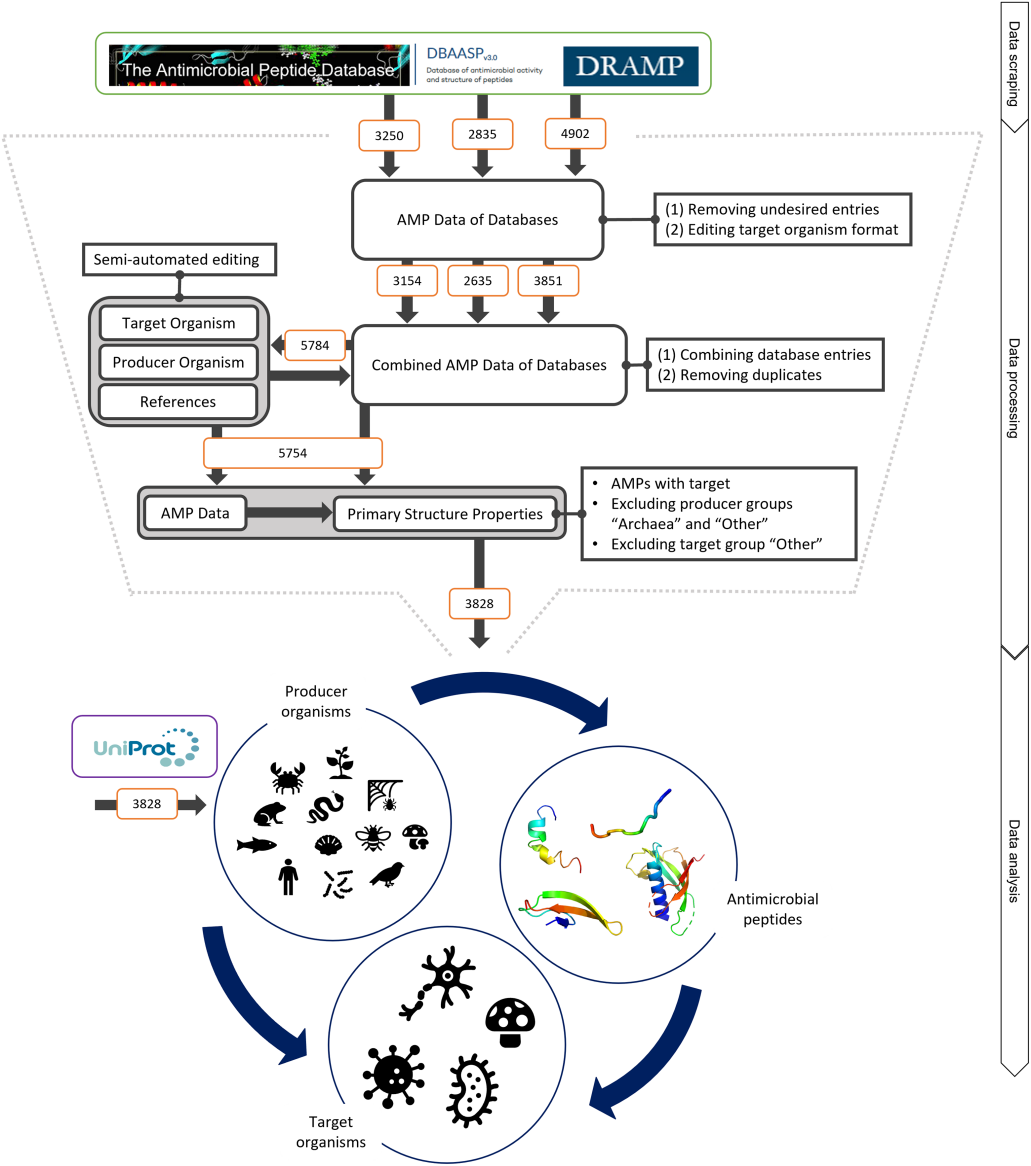
Schematic of data mining, processing and analysis process. Data were retrieved from the three databases APD, DRAMP and DBASSP and subsequently filtered after predefined criteria (no double sequences, only peer-reviewed entries, no synthetic peptides, etc.). This semi-automated editing of the entries improved the data for automated processing. The producer organism group ‘Archaea’ and ‘Other’ as well as the target organism group ‘Other’ were excluded since the number of peptides was not statistically evaluable. This led to total of 3,828 peptides to be analysed. The same amount was retrieved from UniProt to compare the AMP structural features to randomly selected peptides. The peptides were examined regarding (i) the relationship of the producing organism group and the AMP structure, (ii) the dependence of the investigated target organism and the AMP producing group and (iii) the influence of the AMP structure on the target organism. For further details, see text.

The three antimicrobial peptide databases APD, DRAMP and DBAASP (Gogoladze et al., 2014; Wang et al., 2016; Kang et al., 2019) were mined to investigate main structural characteristics of AMPs. In total, 10,987 entries for AMPs were obtained, semi-automatically processed and due to stringent exclusion criteria (e.g. incomplete entries and double entries, see Materials and Methods) reduced to a final number of 3,828 AMPs (Figure 1). Since only the DBAASP database reports if a peptide is of ribosomal or non-ribosomal origin, we did not make a distinction in that point. However, at least 62 AMPs are of non-ribosomal origin, which represents 1.6% of the total analysed AMPs. Thus, the vast majority of analysed peptides is of ribosomal origin. Additionally, inhibiting concentrations are reported in publications and thus in databases in multiple ways [e.g. minimal inhibitory concentration (MIC), inhibitory concentration at 50% (IC50) and hazardous concentration at 50% (HC50)]. As these values depend on the test and cultivation conditions applied,^1^ total values of inhibitory concentrations were not considered in our study.

**Figure 2:**
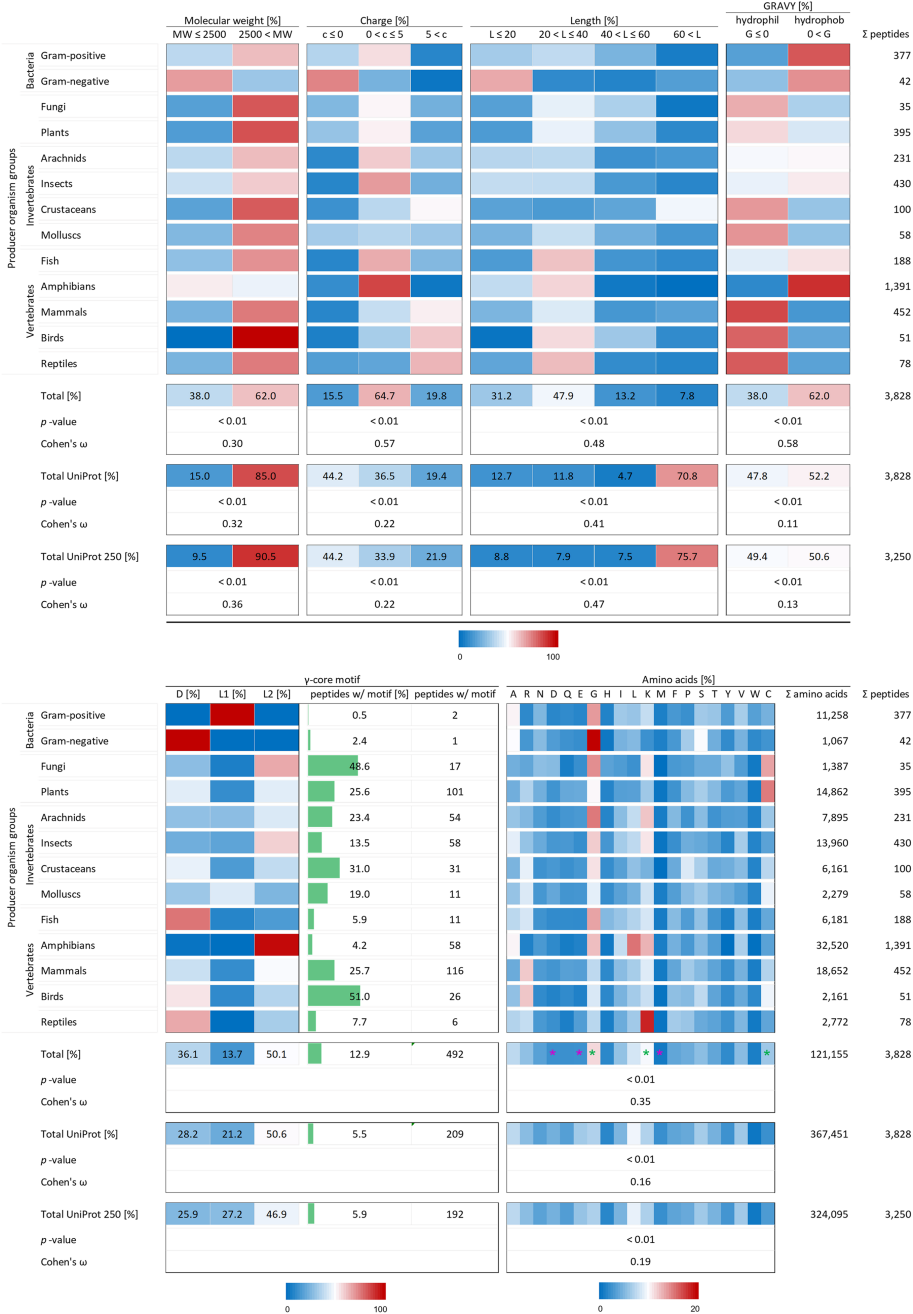
Distribution of AMP properties in different producing organism groups. Data were mined from the APD, DRAMP and DBAASP databases and subsequently processed. Percentage values were used to generate heat maps with blue colour for low and red colour for high values. The margins for the corresponding tables are given directly below the tables. The row ‘Total’ describes the summary of the data of the producer organism rows. The values in the row named ‘Total UniProt’ are from randomly chosen peptides of the peptide database UniProt. The row ‘Total UniProt 250’ displays the results, if the randomly chosen peptides have a total number of 250 for each producing organism group. The association and its magnitude were determined by the *p*-value and the Cohen’s ω, respectively. Asterisks indicate highest changes for increased (green) and decreased (violet) numbers of amino acid residues compared to randomly chosen peptides from UniProt.

Remaining peptide entries were examined with respect to their structural properties and the corresponding groups of producing as well as target organisms. With 1,391 AMPs, the highest number is reported from amphibians (36.3%), whereas only 35 AMPs (0.9%) are reported from fungal origin (Figure 2). Likewise, AMPs from Gram-negative bacteria (42), molluscs (58), birds (51) and reptiles (78) are only lowly represented in the final AMP protein set, clearly demonstrating a large bias towards amphibian AMP resources.

To test the statistical significance of the structural properties of AMPs associated with the producing organism and to determine the effect size of this association, the Χ^2^ test of independence (significance) and the Cohen’s *ω* (effect size) were calculated according to McHugh (2013) and Cohen (1988). Except for the occurrence of the γ-core motif, all structural characteristics could be proven to be associated with the producer organisms (*p*-values < 0.01). The dataset for a statistical analysis of the γ-core motif did not match the mathematical constraints. Data analysis unveiled and confirmed distinctive structural features of AMPs in general but also showed a dependence of structural features on their producing taxonomic group. The investigated AMP characteristics included the molecular weight, the charge at physiological pH (pH 7.4), the length, the hydropathy (GRAVY value), the occurrence of a γ-core motif and the amino acid composition. At least 62% of the investigated 3,828 AMPs showed a molecular weight higher than 2,500 Da, a net positive charge between 0 and + 5, an overall length below 40 amino acids and shared a hydrophobic character (Figure 2) and thus define a common theme. Furthermore, the amino acid composition of the AMPs is enriched in lysine, cysteine and glycine, but depleted in aspartic acid, glutamic acid and methionine when compared to a randomly chosen peptide set from the UniProt database of the same size (Figure 2, compare total values in percent). These findings are statistically significant in all cases (*p*-values < 0.01) and confirm the common description of typical AMP properties reported in most studies (Bondaryk et al., 2017; Huan et al., 2020).

Interestingly, the γ-core motif is present in less than 13% of the 3,828 AMPs, which is still more than double the amount compared to randomly chosen peptides (5.5%; Figure 2, compare total values in percent). Those AMPs having a potential γ-core mainly contain the levomeric L2-isoform (50.1%) or the dextromeric D-isoform (36.1%), whereas the levomeric L1-isoform is less frequent (13.7%, Figure 2). Hence, the γ-core which is described as unifying structural signature present in all cysteine-stabilised AMPs is less abundant than presumed, if considered as fundamental contributor of AMP’s activity. Thus, the activity of the vast majority of AMPs does not depend on the presence of the γ-core motif. However, 9 out of 13 taxonomic groups that produce AMPs show a higher abundance of the γ-core motif in their AMPs compared to the random UniProt set of peptides, whereas four taxonomic groups (Gram-positive, Gram-negative, fish and amphibians) show an equal or even lower abundance. Consequently, these results further corroborate the γ-core motif as characteristic part of several AMPs but also mitigate its importance of being a unifying structural element in this group.

Interestingly, the molecular pattern of AMPs from several AMP producers deviates from the common description summarised above, e.g. from Gram-negative bacteria. This group mainly encodes AMPs with a molecular weight below 2.5 kDa, a net negative charge, the second lowest abundance of a γ-core motif and an increased amount of alanine and serine in their amino acid sequence (Figure 2 and Table 1). Additionally, amphibians also appear to produce rather small AMPs with a molecular weight below 2.5 kDa and possess a higher amount of alanine (Table 1). Further AMPs that deviate from the typical AMP pattern are found in mammals, birds and reptiles. These groups produce AMPs with a net positive charge higher than +5 and with a hydrophilic character (Table 1). Moreover, AMPs from mammals and birds show high amounts of arginine in their amino acid sequences, whereas lysine is below average. Regarding those AMPs containing a γ-core motif, birds (51%) and fungi (48.6%) clearly show much increased presence of this characteristic in their AMPs. Thus, the natural AMP reservoir is more versatile than expected.

X^2^ analysis demonstrated that the analysed AMP properties are statistically significantly related to the producing organism group. We therefore performed Cohen’s ω calculations to evaluate differences in the effect size of these associations (see methods). This led to a clear ranking in the structural characteristics of AMPs (Figure 2). Whereas all structural features show at least small Cohens ω-values (>0.1), these values were high in case of charge (0.57) and hydropathy (0.58). Peptides of the same producing organism group randomly chosen from UniProt only showed a medium effect size (0.22) for the relation of producer and charge and a low effect size for the relation to hydropathy (0.11). Moreover, the effect size for the amino acid composition of randomly selected peptides (0.16) is less than half than of AMPs (0.35). These findings for randomly chosen proteins from UniProt also did not significantly change when an equal amount of 250 peptides was chosen for each producing organism group (Figure 2). This indicates a high-ranking importance of the charge, hydropathy and amino acid composition as determinants of the molecular patterns of AMPs. Even though the previously used statistics could not be applied to the occurrence of a γ-core motif, its sequence was found 2.3-fold more often in AMPs (12.9%) than in the randomly selected peptide control group (5.5%). In contrast, the molecular weight and length of AMPs are less important when compared to randomly selected peptides within a producing organism group.

Taken together, these data demonstrate tendencies of structural properties for each producer group even though uniform specific molecular characteristics can be found for the majority (62%) of AMPs.

**Figure 3:**
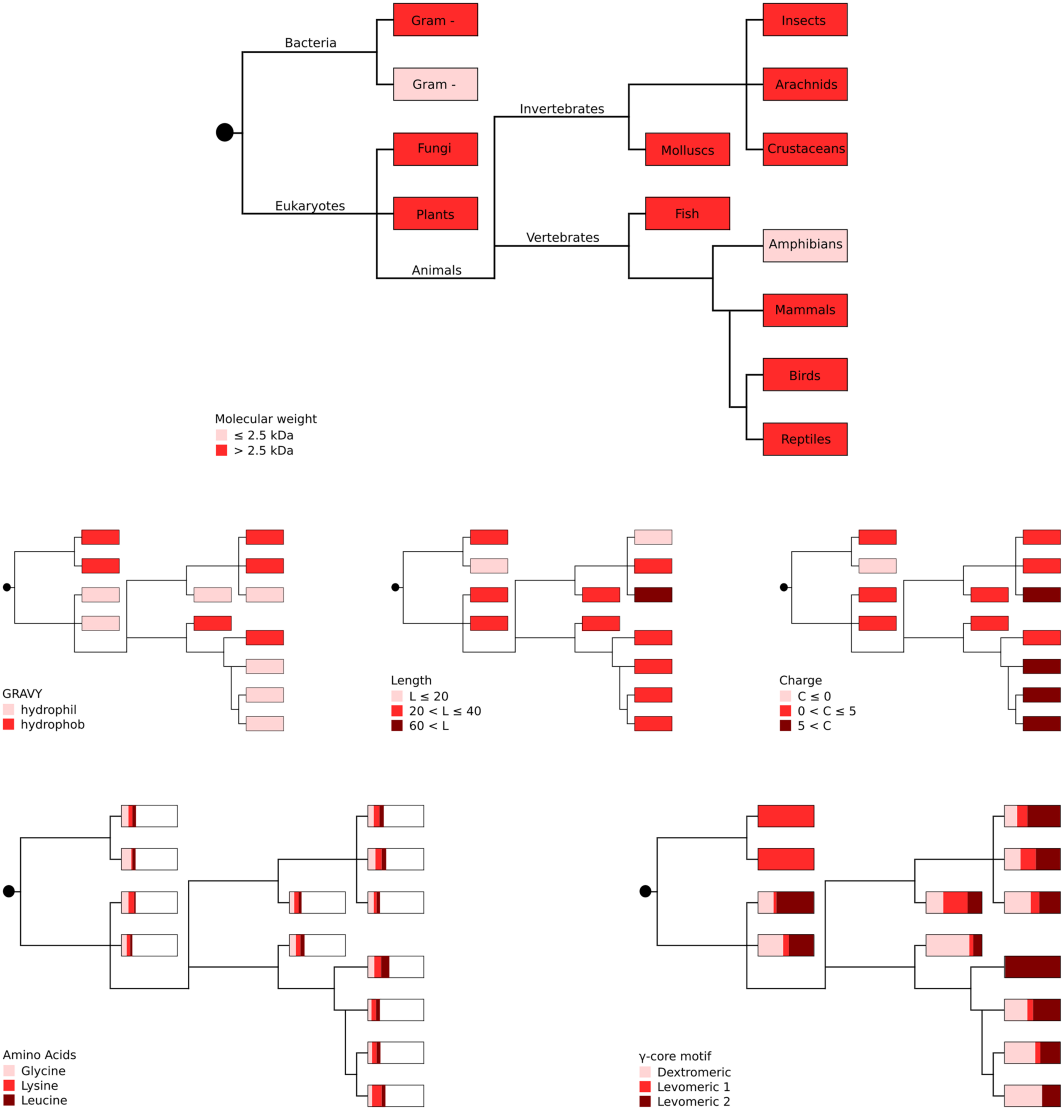
Phylogenetic representation of the most abundant AMP properties per producing organism group. The most abundant amino acids (glycine, lysine and leucine) as well as the different γ-core motifs are given as proportions with boxes representing 100%. Gram + represents Gram-positive and gram – represents Gram-negative bacteria. Distribution of the taxonomic groups is as labelled in the top panel. Each phylogenetic tree has its own colour legend and is related to one of the following AMP properties (as stated in the figure): molecular weight, GRAVY, length, charge, amino acids and γ-core motif.

Our *in silico* analyses did not unveil a phylogenetic connection between the producing organism group and the occurrence of specific AMP properties (Figure 3), suggesting high and independent adaptive variation of AMPs throughout evolution with their functional properties being pronounced to a greater or lesser extent.

### AMP Activity Tests Have a Strong Bias Towards Specific Target Organisms

**Figure 4:**
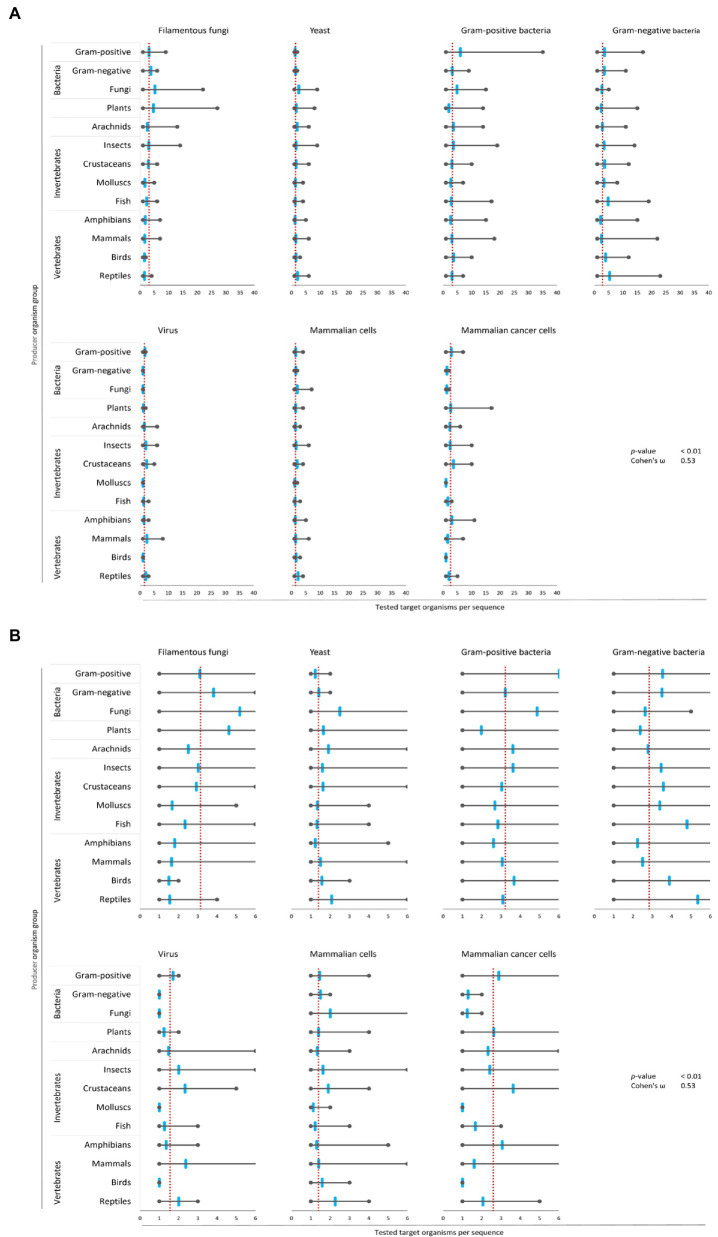
Bias of AMP activity testing. Data were mined from the APD, DRAMP and DBAASP databases and subsequently processed. The average of tested species for each target group was determined (red dotted line). Additionally, the mean (blue mark), the maximum (right grey mark) and the minimum (left grey mark) of tested species for each target organism and producing organism group were calculated. For better assessment of the displayed data, the diagrams of **(A)** were magnified and are displayed in **(B)**. The association and its magnitude were determined by the *p*-value and the Cohen’s ω, respectively.

Next, we calculated for each target organism group the mean, the maximum and the minimum of tested species for all 3,828 AMPs. Subsequently, these values were used to determine if target organisms were tested to a greater or lesser extent per producing organism group than the average of all AMPs of each target organism group. This approach uncovered that bacteria were tested for the highest number of AMPs (Gram-positive: 3,209 peptides, Gram-negative: 3,080 peptides), followed by yeast (1,708 peptides) and mammalian cells (1,449 peptides). Filamentous fungi (705 peptides), mammalian cancer cells (465 peptides) and viruses (209 peptides) were the least tested target organism groups (Figure 4).

The average amount of species tested per AMP was the highest for Gram-positive bacteria (3.2 target organisms per AMP) and filamentous fungi (3.1 target organisms per AMP), followed by Gram-negative bacteria (2.8 target organisms per AMP) and mammalian cancer cells (2.6 target organisms per AMP, Figure 4). Hence, independent of the AMP source activity testing against bacteria comprises a broader set of tested bacterial species than filamentous fungi, yeast, virus or cancer cells. In contrast, yeast, virus and mammalian cells were tested with 2–3 times lesser extent (1.3–1.5 target organisms per AMP). Assessing the single producing organism groups, peptides from fungi are tested above average regarding their activity towards filamentous fungi, yeast and Gram-positive bacteria (Figure 4). However, AMPs produced by plants are most often tested towards their activity against filamentous fungi and yeast, but below average against Gram-positive, Gram-negative and viruses. Activity testing of AMPs from amphibians and mammals is less often performed against filamentous fungi, Gram-positive and Gram-negative bacteria.

These results show an association (*p*-value <0.01) with a high effect size (Cohen’s *ω* = 0.53), clearly showing a biased choice of tested target organisms that depends on the AMP producer organism group. Hence, the evaluation of the relationship between the AMPs’ structural characteristics and a target organism group is likely compromised by the bias in AMP activity testing.

### AMPs Directed Towards Specific Human Pathogens Show Distinct Structural Properties

Although a bias was detected regarding the producer organism group of AMPs and the target organisms tested, two fungal species and ESKAPE bacteria were examined to evaluate possible structure-target organism relationships. Again, the investigated AMP molecular characteristics included the molecular weight, the charge at physiological pH (pH 7.4), the peptide length, the hydropathy (GRAVY), the occurrence of a γ-core motif and the amino acid composition.

**Figure 5:**
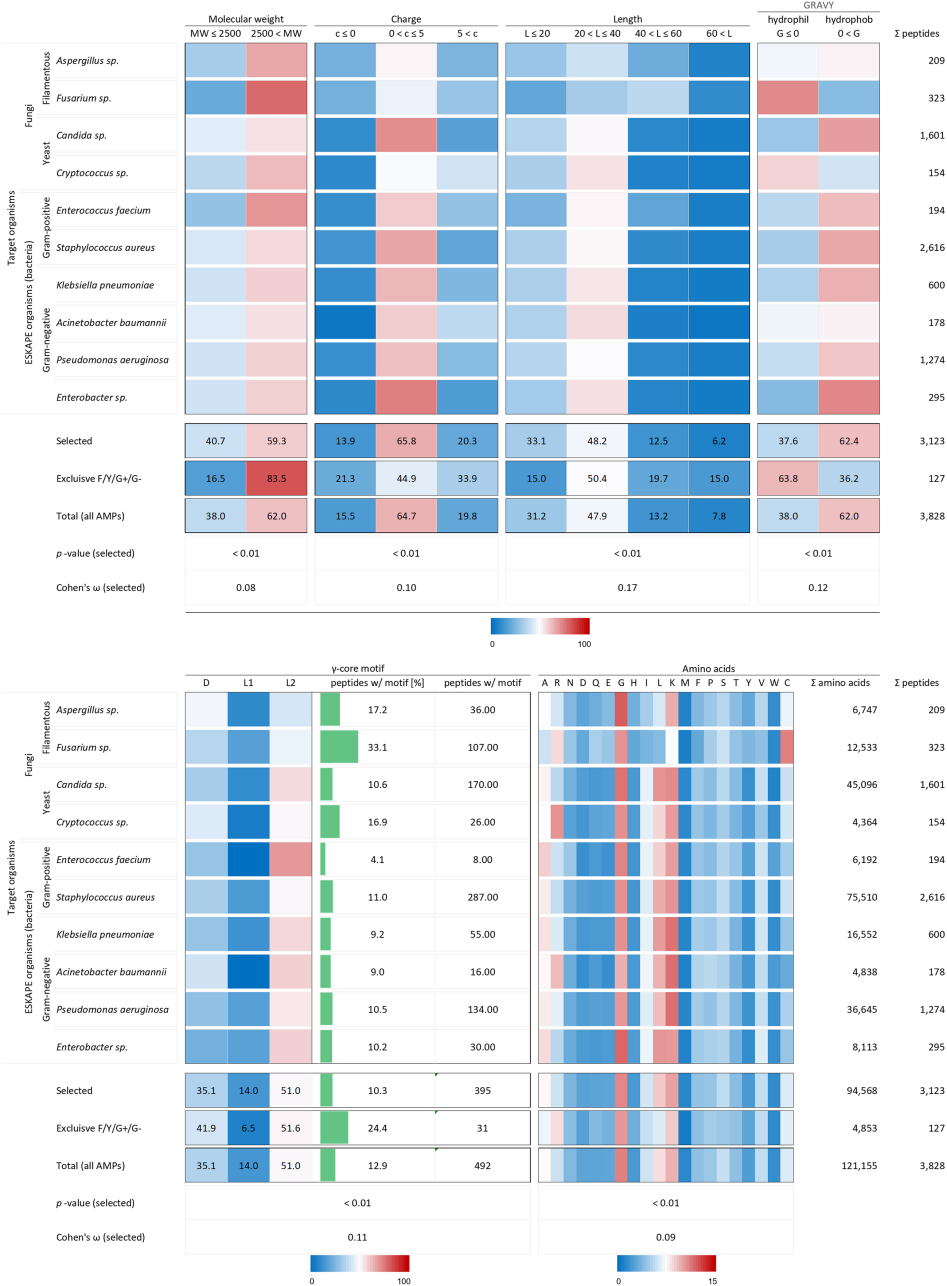
Distribution of AMP properties in different target organism groups. Data were mined from the APD, DRAMP and DBAASP databases and subsequently processed. Percentage values were used to generate heat maps. The row ‘Total’ describes the summary of the data of the target organism rows. The values in the row named ‘Exclusive F/Y/G+/G-’ summarise the properties from AMPs, which encompass in parallel activities against filamentous fungi (F), yeast (Y), Gram-positive (G+) and Gram-negative (G-) bacteria. The row ‘Total (all AMPs)’ displays the results when all AMPs are considered (for comparison, see Figure 2). The association and its magnitude were determined by the *p*-value and the Cohen’s ω, respectively.

All analysed 3,123 AMP structural characteristics were statistically significantly associated with the investigated target organisms (*p* < 0.01). However, the strength of the relationship, expressed as Cohen’s *ω*, was low for the charge (0.10), the length (0.17) and the γ-core motif (0.12) and very low for the molecular weight (0.08) and the amino acid composition (0.09, Figure 3). AMPs examined against the chosen target organisms are generally bigger than 2.5 kDa, have a charge between 0 and +5, a length between 20 and 40 amino acids are hydrophobic and possess the levomeric L2-isoform of the γ-core motif (Figure 5). This pattern is equivalent to the overall molecular pattern of characteristics of all examined AMPs (Figures 2, 5). However, AMPs with concurrent activities against fungi, Gram-positive and Gram-negative bacteria had varying strength of the occurrence of these characteristics. Those 127 AMPs showed a larger molecular weight (83.5%) and length, a more diffuse net charge, are more often hydrophilic (63.8%), have a higher content of the γ-core motif (24.4%), and showed deviations in their amino acid sequence with more arginine and histidine and less serine and leucine compared to the average AMP molecular pattern. Interestingly, at least 1.5-fold more AMPs with activities against *Aspergillus* spp., *Fusarium* spp. and *Cryptococcus* spp. Contained the γ-core motif (16.9–33.1%) compared to AMPs with activities against the ESKAPE organisms (4.1–11%). Additionally, AMPs with activities against *Fusarium* spp. contained a comparatively high amount of cysteine residues in parallel to the γ-core motif with a higher abundance, and they were more often hydrophilic than the other AMPs. Notably, 2,616 of all investigated AMPs (3,828) were tested against the organisms *Staphylococcus aureus* (68.3%), followed by 1,601 AMPs against *Candida* sp. (41.8%) and 1,274 AMPs against *Pseudomonas aeruginosa* (33.3%). This further corroborates the observation of a bias in AMP activity testing.

Taken together, these results show a clear variation of the AMPs structural properties related to their activities against specific (*Fusarium* spp. Or *Cryptococcus* spp.) or a broader range of pathogens (filamentous fungi, yeast and bacteria) compared to AMPs which do not show a corresponding activity. However, the data can only deliver a limited insight regarding the relation between the AMPs properties and their effects on target organisms due to the bias in activity testing.

## Discussion

The natural AMP reservoir is more diverse than anticipated – the results of this study clearly show specific structural properties of AMPs that are distinct from randomly chosen peptides. The most common description of AMPs includes a molecular weight above 2.5 kDa, a length below 40 amino acid residues and a hydrophobic character with a net positive charge between 0 and + 5 (Figure 2). Although these AMPs properties are true for at least 62% of all AMPs analysed in this study, up to 38% of the remaining AMPs differ from this generalisation. The latter AMP group comprises a hydrophilic character, a negative or strongly increased net positive charge above +5 or a length of more than 40 amino acid residues.

An additional important feature is the specific amino acid profile of AMPs including increased amounts of cysteines, lysines and glycines, whereas amounts of methionine, aspartate and glutamate residues are decreased compared to randomly selected peptides from UniProt. However, AMPs from some producer organism groups differ from this description, e.g. Gram-negative bacteria have less cysteines and more alanine in comparison with AMPs or randomly selected peptides. Consequently, search strategies using generalised or specific AMP molecular patterns will miss potential AMP candidates if applied to other taxonomic groups. Therefore, future AMP screening programmes mainly based on *in silico* data must consider these two limitations.

The natural AMP reservoir remains largely unexploited – our survey uncovered a large bias in AMP research. More than one-third of analysed AMPs are derived from amphibians, whereas fungi, Gram-negative bacteria, molluscs, birds and reptiles show the lowest numbers of investigated AMPs. They display together less than 7% of all AMPs analysed in this study. Hence, the choice of a target organism to be tested needs to be uncoupled from the AMP producing organism and, ideally, a broader set of target organisms needs to be tested by default. The high clinical relevance of bacterial pathogens is one a reason why research is focussed on antibacterial compounds and the neglection of other pathogens (fungi, yeast and virus) or diseases (cancer cells). However, fungal infections annually kill more patients than tuberculosis or malaria, a severe threat that is not well communicated in public debates compared to bacterial infections (Bongomin et al., 2017). Crucial for future AMP screening programmes are thus the implementation of standardised tests of AMPs against a broad set of microorganisms, insects, parasites and mammalian/cancer cells to leverage the natural AMP reservoir and aid new drug templates in both the clinics and agriculture.

Modular design of AMPs – various AMPs evolved in organisms from different habitats, leading to a wide variety of AMP structures and antimicrobial activities. Using the obtained data of the three databases, we could associate multiple structural characteristics of AMPs to their producing organism group. Interestingly, only the charge, the hydropathy (GRAVY), and the amino acid fingerprint of AMPs were found to be stronger related to the peptide producing group than in randomly chosen control peptides of the same producing group. Furthermore, the amino acid composition differs greatly among the producing organism group although they follow a general AMP structural pattern. In contrast, the molecular size given in length or weight appears to be of less importance for AMPs’ structure definition and activity.

**Figure 6:**
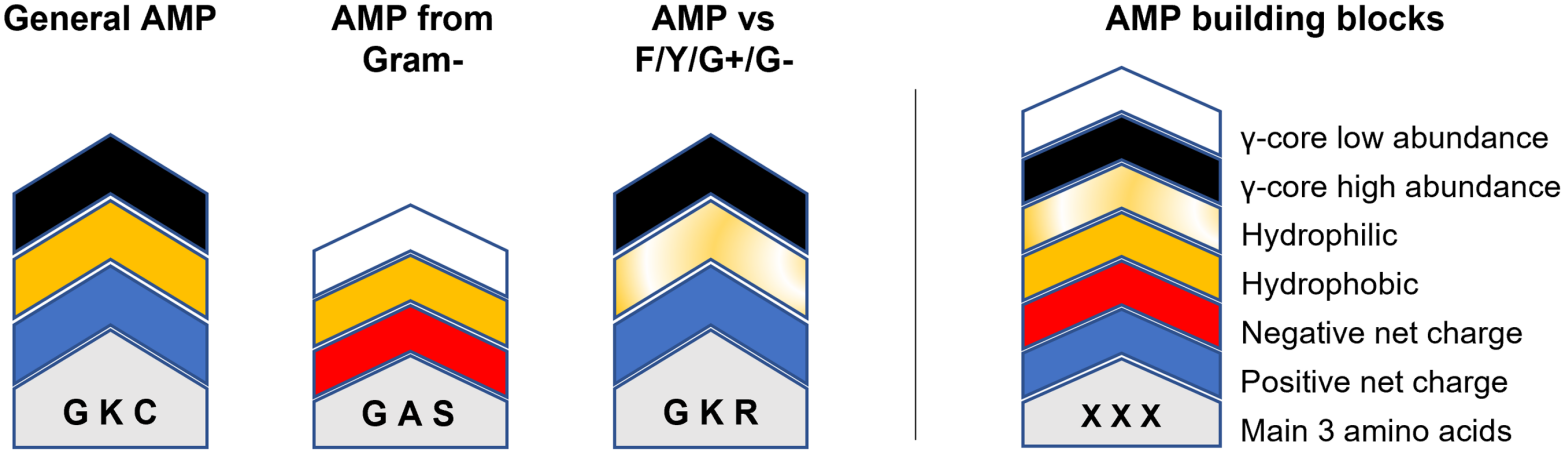
Modular AMP design approach. Relevant building blocks determining the structural properties of AMPs are given on the right. The molecular weight/length is indicated by the overall height of the building block stacks on the left which can be either higher or lower 2.5 kDa/20 residues. Amino acids are indicated by one-letter code. ‘General AMP’ displays the average structure retrieved from all analysed AMPs. ‘AMP from gram-’displays the average structure of all AMPs produced exclusively by Gram-negative bacteria. ‘AMP vs. F/Y/G+/G-’shows the main structural properties of AMPs with concurrent activities against filamentous fungi (F), yeast (Y), Gram-positive (G+) and Gram-negative (G-) bacteria.

Could this knowledge already be used to aid AMP design? For example, AMPs targeting *Fusarium* spp. or *Cryptococcus* spp. show an accumulation of the γ-core motif are rather hydrophilic than hydrophobic and include rather arginine than lysine residues (Figure 5). Additionally, they have high amounts of cysteine residues, in the case of AMPs tested against *Fusarium* spp. (Figure 5). In contrast, AMPs targeting the ESKAPE pathogen *Enterococcus faecium* appear to be rather hydrophobic than hydrophilic, include rather lysine than arginine residues, contain increased amounts of alanine residues and show less accumulation of the γ-core (Figure 5). However, can AMPs with broad-spectrum antimicrobial activities towards fungi, yeast and bacteria be designed based on their structural deviations from the generalised structural pattern? Figure 6 highlights the concept of a building block approach that envisions a future modular design of (semi)synthetic AMPs to combat microbial infections and diseases.

Still, the data input for this concept is very limited and biased and thus need to be applied cautiously. For example, although AMPs with an activity towards *Fusaria* or *Cryptococci* show the γ-core as structural property, this does not imply that AMPs having a γ-core are automatically active against these target organisms.

## Conclusion

Due to inconsistent entries in the antimicrobial peptide databases APD, DRAMP and DBAASP, 3,828 AMPs out of 10,987 AMPs were available for the current study. We could show that a correlation between the producer organism of an AMP, its structural molecular pattern and the target organism exists. The presented data support the general assumption that natural AMPs can provide scaffolds for the development of novel antimicrobial compounds, which might be applied in the clinics and agriculture. To fully exploit that natural potential, a standardised testing and reporting method regarding the AMPs activity towards target organism and target molecular structures needs to be established or at least followed. The European Committee on Antimicrobial Susceptibility Testing (EUCAST) and the US Clinical and Laboratory Standards Institute (CLSI) provide corresponding standard protocols^2^^,^^3^, which represent a gold standard, ensuring the avoidance of research bias and an acceleration of the development of novel antimicrobial agents.

## Data Availability Statement

The original contributions presented in the study are included in the article/Supplementary Material; further inquiries can be directed to the corresponding author.

## Author Contributions

CF performed the data mining, the processing and statistical analysis of the retrieved data. CF and SJ developed the study design and coordinated the project. CF, SJ, and VM performed the data interpretation and wrote the manuscript. All authors have read and approved the final manuscript.

## Funding

The work was funded by the Deutsche Forschungsgemeinschaft (DFG, GRK2473 ‘Bioactive Peptides’—project number 392923329). We acknowledge support by the Deutsche Forschungsgemeinschaft, the Open Access Publication Funds of TU Berlin, and the German DEAL consortium for open access funding.

## Conflict of Interest

The authors declare that the research was conducted in the absence of any commercial or financial relationships that could be construed as a potential conflict of interest.

## Publisher’s Note

All claims expressed in this article are solely those of the authors and do not necessarily represent those of their affiliated organizations, or those of the publisher, the editors and the reviewers. Any product that may be evaluated in this article, or claim that may be made by its manufacturer, is not guaranteed or endorsed by the publisher.

## Supplementary Material

The Supplementary Material for this article can be found online at https://www.frontiersin.org/articles/10.3389/fmicb.2022.812903/full#supplementary-material

Supplementary Additional File 1 Detailed description of the data mining and processing process.

Supplementary Additional File 2 Mined data from the APD website.

Supplementary Additional File 3 Mined data from the DRAMP website.

Supplementary Additional File 4 Mined data from the DBAASP website.

Supplementary Additional File 5 Final processed AMP data.

Supplementary Additional File 6 Primary structure characteristics of the peptides in Additional File 5.

Supplementary Additional File 7 Final UniProt peptides.

Supplementary Additional File 8 Primary structure characteristics of the control peptides in Additional File 7 with an equal amount as test producer groups.

Supplementary Additional File 9 Primary structure characteristics of the control peptides in Additional File 7 with 250 peptides per producer groups.

## Footnotes

^1^www.eucast.org, Accessed: November 9th, 2021.

^2^www.eucast.org, Accessed: November 9th, 2021.

^3^www.clsi.org, Accessed: November 9th, 2021.

## Abbreviations

AMPs: Antimicrobial peptides.
APD Antimicrobial peptide database.
DBAASP: Database of antimicrobial activity and structure of peptides.
DRAMP: Data repository of antimicrobial peptides
