# Supplementary material for: Structure–Activity Predictions From Computational Mining of Protein Databases to Assist Modular Design of Antimicrobial Peptides
Source: Front Microbiol. 2022 Apr 15;13:812903. doi: 10.3389/fmicb.2022.812903 (PMC9075106; doi:10.3389/fmicb.2022.812903)
Supplement: Supplementary Additional File 1 — Detailed description of the data mining and processing process. [file Table_1.DOCX]

This is a description for what have been done on AMP Data mining. All programs and excel files plus sheets are highlighted.

**Database files**

**DRAMP Database** (http://dramp.cpu-bioinfor.org/)

- Data mined: 23^rd^ July 2020
- ID: DRAMP_#
- Data mining program: Files been downloaded on website (http://dramp.cpu-bioinfor.org/downloads/) under "Activity"
  - Folder "DRAMP_Files" combined => DRAMP_Database.xlsx
- DRAMP_Database.xlsx
- original w double IDs (Double IDs had been deleted)
- original (Double IDs had been deleted)

→ ***Further handling

**APD Database** (http://aps.unmc.edu/AP/main.php)

- Data mined: 11^th^ October 2020
- ID: AP_#
- Data mining program: APD_Database.py => ADPDatabase_PY.xlsx
- IDs to be mined received via: AP00001 to AP03250 (sequential number)

=> id_num_APD.xlsx

- ADP_Database_PY.xlsx
- original

→ ***Further handling

**PD Database** (https://dbaasp.org/)

- Data mined: 23^rd^ July 2020
- ID: PD_#
- Data mining program: DBAAS_Database.py => DBAASDatabase_PY.xlsx
- IDs to be mined received via:

1. Look up producer IDs: https://dbaasp.org/api/v1?query=lookup&lookup_type=kingdom&format=xml
2. use: https://dbaasp.org/api/v1?query=search&kingdom_id=*****&format=xml (*****: kingdom ID: 42, 45, 52, 53, 54, 268 standing for Animalia, Fungi, Bacteria, Plantae, Protista, Virus, respectively)
3. save IDs listed in each kingdom ID → id_num_PD.xlsx

- DBAAS_Database_PY.xlsx
- original

→ ***Further handling

*** **Further handling**

similar Sheets in Excel files DRAMP_Database.xlsx, ADPDatabase_PY.xlsx and DBAASPDatabase_PY.xlsx:

- IDs are marked blue, of which sequences including an X (= non-canonical amino acid)
- marked X
- IDs are deleted, of which sequences including an X (= non-canonical amino acid)

IDs are marked blue, which don’t have a publication given, also "submitted" or thesis have been not considered a publication

- wo X+marked pub
- IDs are deleted, which don’t have a publication given

IDs are marked blue, which have a synthetic producer

- wo pub+marked syn prod
- IDs are deleted, which have an synthetic producer

Targets are edited after the following pattern: “Organism, further specification like strain, inhibition concentration; “ Not included, but collected in separate column are undefined organisms (bacteria, Gram+, Gram-, haemolytic etc.) and expressly indicated as no activity

DBAASP: OrganismFindDBAASP.py

DRAMP + APD: edited manually

- wo syn prod + processed targ

**Main working file**

CombinedAMPData.xlsx

- Data from file APD_Database_PY.xlsx, wo syn prod + targ manual edit
- APD data edited
- Data from file DBAASP_Database_PY.xlsx, wo syn prod + targ manual edit
- DBAASP data edited
- Data from file DRAMP_Database.xlsx, wo syn prod + targ manual edit
- DRAMP data edited
- Combination of the columns (ID, Sequence, Length, Name, Producer, Target, No activity, Undefined, Reference) all three databases
- Combined Data (Data of all three databases in a row)
- Finding related sequences

1. Sort data by sequence
2. Rules if more than one ID of a database for one sequence:

- Do they have different entry IDs + C or N terminal modification?

→ Yes: different sequence + check the other IDs if they also have a modification + appropriate allocation;

- Do they have different entry IDs but else is all the same? → Yes: same sequence
- Do they have different entry IDs + different reference but else is all the same? → Yes: same sequence
- Do they have different entry IDs + different name + else is all the same/reference different? → Yes: different sequence

→ In case of doubt original websites has been rechecked using ID

→ The differentiated sequences been added at the end of the list

1. Check which ID is the last for the sequence and mark it (This are the sequences which are kept)
2. Merge rows of sequences considered as the same (all columns except of Sequence and Length were joined)

- Seq Sorted + marked
- Delete rows which are not marked as “Last”
- Seq joined + only "Last"
- IDs marked which have no producer OR multiple genus per multiple IDs not class
- invalid prod marked
- Marked IDs deleted
- wo invalid prod

**Helper files**

**ProducerOrganism.xlsx**

- Producer column from CombinedAMPData.xlsx, Sheet ...)
- original
- Remove double: =AND(ISTEXT(D2), COUNTIF(2:2,D2)>1),

marked ID: no producer if multiple producers, not the same class

organisms chosen for cohorts:

Only one ID → first organism

Synonym? Yes → choose one organism

Same class? Yes → choose one organism

- wo double, manual edit
- Marked IDs deleted
- wo double+marked, manual edit
- ProducerOrganism.py and manual assignment to cohorts
- organism cohorts

**TargetOrganism.xlsx**

- Target column from CombinedAMPData.xlsx, prod edited + invalid marked
- original
- Finding easy to eradicate mistakes using TargetOrganism_1.py
- target list original
- original without easy to eradicate mistakes
- original (2)
- TargetOrganism_1.py
- target list man edit
- Old: =TRIM() + Remove duplicates
- New: Genus and species (if given) name of target in column Old manually edited using NCBI taxonomy browser and Google
- Cohort: Manual assignment using NCBI taxonomy browser
- TargetOrganism_2.py
- target organisms edited
- TargetOrganism_2.py
- target list cohorts

**References.xlsx**

- Reference column from CombinedAMPData.xlsx, wo invalid prod + target edited
- original
- Manual removing of double references
- manual edited references

**Result files** (“raw data”) for analysis

**AMPsData.xlsx**

- Merge data of CombinedAMPData.xlsx, wo invalid prod + target edited (1), ProducerOrganismus.xlsx, organism cohorts (2), TargetOrganism.xlsx, target organisms edited (3), TargetOrganism.xlsx, target list cohorts (4) and References.xlsx, manual edited references (5)
- AMP_List

1. ID, Sequence, Length, Name, Producer, No activity, Undefined
2. Producer cohort
3. Target
4. Target cohorts
5. References

**PrimarySequenceProperties.xlsx**

- GRAVY.py
- GRAVY
- GammaCoreMotif_WS_D.py, GammaCoreMotif_WS_L1.py, GammaCoreMotif_WS_L2.py and GammaCoreMotifValidation.py
- Gamma Core Motif
- ProteinCalculator.py
- pI + mol.weight + charge pH 7.4
- ProteinCalculator.py
- AA Residues

**UniProt Data** (https://www.uniprot.org/uniprot/#orgViewBy)

- Data mined: 2^nd^ December 2020
- min 20 Results per phylum, phylum from ProducerOrganism.xlsx
- Gram_positive.xlsx (Gram_pos_1.xlsx, Gram_pos_2.xlsx, Gram_pos_3.xlsx)

(1) taxonomy:"Actinobacteria [201174]" AND reviewed:yes

(2) taxonomy:"Firmicutes [1239]" AND reviewed:yes

(3) taxonomy:"Deinococci [188787]" AND reviewed:yes

- - Gram_negative.xlsx (Gram_neg_1.xlsx, Gram_neg_2.xlsx, Gram_neg_3.xlsx, Gram_neg_4.xlsx)

(1) taxonomy:"Cyanobacteria [1117]" AND reviewed:yes

(2) taxonomy:"Proteobacteria [1224]" AND reviewed:yes

(3) taxonomy:"Spirochaetes [203691]" AND reviewed:yes

(4) taxonomy:"Thermotogae [200918]" AND reviewed:yes

- - Fungi.xlsx

taxonomy:"Fungi [4751]" AND reviewed:yes

- Plants.xlsx

taxonomy:"Viridiplantae [33090]" AND reviewed:yes

- Arachnida.xlsx

taxonomy:"Arachnida [6854]" AND reviewed:yes

- Insects.xlsx

taxonomy:"Dicondylia [85512]" AND reviewed:yes

- Crustaceans.xlsx

taxonomy:"Crustacea [6657]" AND reviewed:yes

- Molluscs.xlsx

taxonomy:"Mollusca [6447]" AND reviewed:yes

- Fish.xlsx (Fish_1.xlsx, Fish_2.xlsx, Fish_3.xlsx, Fish_4.xlsx)

(1) taxonomy:"Hyperoartia [117569]" AND reviewed:yes

(2) taxonomy:"Dipnoi (lungfishes) [7878]" AND reviewed:yes

(3) taxonomy:"Chondrichthyes [7777]" AND reviewed:yes

(4) taxonomy:"Actinopterygii [7898]" AND reviewed:yes

- Amphibians.xlsx

taxonomy:"Amphibia [8292]" AND reviewed:yes

- Mammals.xlsx

taxonomy:"Mammalia [40674]" AND reviewed:yes

- Birds.xlsx

taxonomy:"Aves [8782]" AND reviewed:yes)

- Reptiles.xlsx (Reptiles_1.xlsx, Reptiles_2.xlsx, Reptiles_3.xlsx)

(1) taxonomy:"Crocodylia (alligators and others) [1294634]" AND

reviewed:yes

(2) taxonomy:"Testudines (turtles) [8459]" AND reviewed:yes

(3) taxonomy:"Lepidosauria (lepidosaurs) [8504]" AND reviewed:yes

- Choosing random entries (max. length: 190 = longest peptide in AMPs)
  - Give random number: =RAND()
  - Choose random entry:

=INDEX(*range entry number*, RANK(*random number in that row*, *range random number*), 1)

1. According to peptide amount of AMPs per producer cohort

(Gram+: 377, Gram-: 42, Fungi: 35, Plants: 395, Arachnida: 231, Insects: 430, Crustaceans: 100, Molluscs: 58, Fish: 188, Amphibians: 1391, Mammals: 452, Birds: 51, Reptiles: 78)

**PrimarySequenceProperties_UniProt.xlsx**

- GRAVY.py
- GRAVY
- GammaCoreMotif_WS_D.py, GammaCoreMotif_WS_L1.py, GammaCoreMotif_WS_L2.py and GammaCoreMotifValidation.py
- Gamma Core Motif
- ProteinCalculator.py
- pI + mol.weight + charge pH 7.4
- ProteinCalculator.py
- AA Residues

1. 250 random peptides per producer cohort

**PrimarySequenceProperties_UniProt_250.xlsx**

- GRAVY.py
- GRAVY
- GammaCoreMotif_WS_D.py, GammaCoreMotif_WS_L1.py, GammaCoreMotif_WS_L2.py and GammaCoreMotifValidation.py
- Gamma Core Motif
- ProteinCalculator.py
- pI + mol.weight + charge pH 7.4
- ProteinCalculator.py
- AA Residues

**Python source codes:**

Source codes generated using Python 3.8 in the IDE PyCharm 2020.1.1 and the website http://www.planetb.ca/syntax-highlight-word

1. **import** openpyxl
2. **from** openpyxl **import** Workbook
3. **import** requests
4. **from** bs4 **import** BeautifulSoup
5. **import** re
6. **from** tqdm **import** tqdm
8. wbExelNeu = Workbook()
9. wbExcel = openpyxl.load_workbook('repository')
10. wsExcel = wbExcel['IDs']
12. url = 'http://aps.unmc.edu/AP/database/query_output.php?ID={}'
14. session = requests.Session()
16. **for** row **in** tqdm(range(2, 3252)):
17. ID = wsExcel['A{}'.format(row)].value[2:]
18. page = session.get(url.format(ID), headers=browserHeaders)
19. soup = BeautifulSoup(page.content, 'html.parser')
21. #   AdditionalInfo = TargetOrganims
22. soup1 = soup.prettify(formatter=None)
23. soup2 = soup1.split('Additional info:')
24. soup3 = soup2[1].split('<td width="61%">')
25. soup4 = soup3[1].split('</td>')
26. AdditionalInfo = str(soup4[0].encode('ascii', errors='ignore'))
27. ###############################################################################
29. lines = str(soup).split('\n')
31. #   ID
32. control = False
33. **for** i **in** lines:
34. **if** control:
35. IDnumber = i[19:-9]
36. **if** 'APD ID' **in** i:
37. control = True
38. **else**:
39. control = False
40. ###############################################################################
41. #   Peptide name
42. control = False
43. **for** i **in** lines:
44. **if** control:
45. ProteinName = i[19:-9]
46. **if** 'Name/Class' **in** i:
47. control = True
48. **else**:
49. control = False
50. ###############################################################################
51. #   SourceOrganism
52. control = False
53. **for** i **in** lines:
54. **if** control:
55. SourceOrganism = i[19:-9]
56. sourceHelper = re.sub("^.*</a>", "", SourceOrganism)
57. sourceHelper1 = sourceHelper.replace("<i></i>", "")
58. sourceHelper2 = re.sub("</i>.*?<i>", ", ", sourceHelper1)
59. sourceHelper3 = re.sub("^.*<i>", "", sourceHelper2)
60. sourceHelper4 = sourceHelper3.split("</i>")
61. **if** 'Source' **in** i:
62. control = True
63. **else**:
64. control = False
65. ##############################################################################
66. #   Sequence
67. control = False
68. **for** i **in** lines:
69. **if** control:
70. Sequence = i[41:-16]
71. **if** 'Sequence' **in** i:
72. control = True
73. **else**:
74. control = False
75. ##############################################################################
76. #   Length
77. control = False
78. **for** i **in** lines:
79. **if** control:
80. Length = i[19:-9]
81. **if** 'Length' **in** i:
82. control = True
83. **else**:
84. control = False
85. ###############################################################################
86. #   Structure
87. control = False
88. **for** i **in** lines:
89. **if** control:
90. Structure = i[19:-9]
91. **if** '3D Structure' **in** i:
92. control = True
93. **else**:
94. control = False
95. ###############################################################################
96. #   Activity
97. control = False
98. **for** i **in** lines:
99. **if** control:
100. Activity = i[19:-9]
101. **if** 'Activity' **in** i:
102. control = True
103. **else**:
104. control = False
105. ##########################################################################
106. #   paperTitle
107. control = False
108. **for** i **in** lines:
109. **if** control:
110. paperTitle = i[19:-9]
111. **if** 'Title' **in** i:
112. control = True
113. **else**:
114. control = False
116. #   paperAuthor
117. control = False
118. **for** i **in** lines:
119. **if** control:
120. paperAuthor = i[19:-9]
121. **if** 'Author' **in** i:
122. control = True
123. **else**:
124. control = False
126. #   paperReference
127. control = False
128. referenceHelper=''
129. **for** i **in** lines:
130. **if** control:
131. ReferenceLine = re.split('<a|</a>]', i)
132. **if** len(ReferenceLine)>1:
133. referenceHelper = ReferenceLine[1]
134. **if** 'Reference' **in** i:
135. control = True
136. **else**:
137. control = False
139. wsExcelNeu = wbExelNeu.active
141. wsExcelNeu['A1'] = 'APD_ID'
142. wsExcelNeu['B1'] = 'Protein_Name'
143. wsExcelNeu['C1'] = 'Source_Organism'
144. wsExcelNeu['D1'] = 'Sequence'
145. wsExcelNeu['E1'] = 'Sequnce_Length'
146. wsExcelNeu['F1'] = 'Activity'
147. wsExcelNeu['G1'] = 'Target_Organism'
148. wsExcelNeu['H1'] = 'paperTitle'
149. wsExcelNeu['I1'] = 'paperAuthor'
150. wsExcelNeu['J1'] = 'Pubmed ID'

153. wsExcelNeu['A{}'.format(row)] = IDnumber
154. wsExcelNeu['B{}'.format(row)] = ProteinName
155. wsExcelNeu['C{}'.format(row)] = sourceHelper4[0]
156. wsExcelNeu['D{}'.format(row)] = Sequence
157. wsExcelNeu['E{}'.format(row)] = Length
158. wsExcelNeu['F{}'.format(row)] = Activity
159. wsExcelNeu['G{}'.format(row)] = AdditionalInfo
160. wsExcelNeu['H{}'.format(row)] = paperTitle
161. wsExcelNeu['I{}'.format(row)] = paperAuthor
162. wsExcelNeu['J{}'.format(row)] = referenceHelper

165. wbExelNeu.save(r'repository')

**Source code 1. APD_Database.py**

1. **import** openpyxl
2. **from** openpyxl **import** Workbook
3. **import** requests
4. **from** bs4 **import** BeautifulSoup
5. **import** re

8. wbExelNeu = Workbook()
9. wbExcel = openpyxl.load_workbook('repository')
10. wsExcel = wbExcel['IDs']
12. url ='https://dbaasp.org/api/v1?query=peptide_card&peptide_id={}&format=xml'
14. session = requests.Session()
16. ActivityHelper = ''
17. PubMedIDHelper = ''
18. PTHelper = ''
20. TOHelper1 = ''
21. TOHelper2 = ''
22. TOHelper3 = ''
23. TOHelper4 = ''
24. TOHelper5 = ''
25. TOHelper6 = ''
27. hemoHelper1 = ''
28. hemoHelper2 = ''
29. hemoHelper3 = ''
30. hemoHelper4 = ''
31. hemoHelper5 = ''
32. hemoHelper6 = ''
34. **for** row **in** range(2, 2837):
35. ID = wsExcel.cell(row, 1).value
36. page = session.get(url.format(ID), headers=browserHeaders)
37. soup = BeautifulSoup(page.content, 'html.parser')
38. lines = str(soup).split('\n')
40. ###############################################################################
41. #   ID
42. IDnumber = ''
43. **for** i **in** lines:
44. **if** '<id>' **in** i:
45. IDnumber = i[4:-5]
46. **print**(IDnumber)
48. #   Sequence
49. Sequence = ''
50. **for** i **in** lines:
51. **if** '<seq>' **in** i:
52. Sequence = i[5:-6]
54. #   Sequence Length
55. SequenceLength = ''
56. **for** i **in** lines:
57. **if** '<seqlength>' **in** i:
58. SequenceLength = i[11:-12]
60. #   Gene
61. Gene = ''
62. **for** i **in** lines:
63. **if** '<gene>' **in** i:
64. Gene = i[6:-7]
66. #   Peptid Name
67. peptideName = ''
68. **for** i **in** lines:
69. **if** '<name>' **in** i:
70. peptideName = i[6:-7]
72. #   Producer Organism
73. producerOrganism = ''
74. **for** i **in** lines:
75. **if** '<source/>' **in** i:
76. producerOrganism += i[9:]
78. #   Target Organism
79. control = False
80. targetOrganism = ''
81. **for** i **in** lines:
82. **if** control **and** '<targetspecies>' **in** i:
83. TOHelper1 += str(i)[15:-16]
84. **if** control **and** '<activitymeasure>' **in** i:
85. TOHelper2 += str(i)[17:-18]
86. **if** control **and** '<concentration>' **in** i:
87. TOHelper3 += str(i)[15:-16]
88. **if** control **and** '<unit>' **in** i:
89. TOHelper4 += str(i)[6:-7]
90. **if** '<targetactivities>' **in** i:
91. control = True
92. **if** '</targetactivity>' **in** i:
93. TOHelper5 = TOHelper1+', '+TOHelper2+'='+TOHelper3+TOHelper4+'; '
94. TOHelper6 += TOHelper5
95. TOHelper1 = ''
96. TOHelper2 = ''
97. TOHelper3 = ''
98. TOHelper4 = ''
99. TOHelper5 = ''
100. **elif** '</targetactivities>' **in** i:
101. control = False
103. # Hemolytic
104. control = False
105. targetOrganism = ''
106. **for** i **in** lines:
107. **if** control **and** '<targetcell>' **in** i:
108. hemoHelper1 += str(i)[12:-13]
109. **if** control **and** '<lysis>' **in** i:
110. hemoHelper2 += str(i)[7:-8]
111. **if** control **and** '<concentration>' **in** i:
112. hemoHelper3 += str(i)[15:-16]
113. **if** control **and** '<unit>' **in** i:
114. hemoHelper4 += str(i)[6:-7]
115. **if** '<hemoliticcytotoxicactivities>' **in** i:
116. control = True
117. **if** '</hemoliticcytotoxicactivity>' **in** i:
118. hemoHelper5 = hemoHelper1+', '+hemoHelper2+'='+hemoHelper3+hemoHelper4+'; '
119. hemoHelper6 += hemoHelper5
120. hemoHelper1 = ''
121. hemoHelper2 = ''
122. hemoHelper3 = ''
123. hemoHelper4 = ''
124. hemoHelper5 = ''
125. **elif** '</hemoliticcytotoxicactivities>' **in** i:
126. control = False
127. Probe = TOHelper6 + '; ' + hemoHelper6
128. targetOrganism = str(Probe.encode('ascii', errors='ignore'))
129. TOHelper6 = ''
130. hemoHelper6 = ''
132. #   Activity
133. control = False
134. **for** i **in** lines:
135. **if** control **and** '<string>' **in** i:
136. ActivityHelper += str(i)[8:-9] + ', '
137. **if** '<targetgroups>' **in** i:
138. control = True
139. **elif** '</targetgroups>' **in** i:
140. control = False
141. Activity = str(ActivityHelper.encode('ascii', errors='ignore'))
142. ActivityHelper = ''
144. #   Swiss-/Uni-Prot ID
145. uniProtID =''
146. **for** i **in** lines:
147. **if** '<uniprot>' **in** i:
148. uniProtID = i[9:-10]
150. #   PDB ID
151. PDB = ''
152. **for** i **in** lines:
153. **if** '<pdb>' **in** i:
154. PDBHelper = re.split('$|</pdb>', i)
155. PDB = PDBHelper[0][5:9]
157. #   PubMed ID
158. control = False
159. **for** i **in** lines:
160. **if** control **and** '<pubmed>' **in** i:
161. PubMedIDHelper += str(i)[8:-9] + ', '
162. **if** '<articles>' **in** i:
163. control = True
164. **elif** '</articles>' **in** i:
165. control = False
166. PubMedID = PubMedIDHelper
167. PubMedIDHelper = ''
169. #######################################################################
170. #   Publication Title
171. control = False
172. **for** i **in** lines:
173. **if** control **and** '<title>' **in** i:
174. PTHelper += str(i)[7:-8] + '; '
175. **if** '<articles>' **in** i:
176. control = True
177. **elif** '</articles>' **in** i:
178. control = False
179. publicationTitle = PTHelper
180. PTHelper = ''

183. ########################################################################
184. wsExcelNeu = wbExelNeu.active
186. wsExcelNeu['A1'] = 'PD_ID'
187. wsExcelNeu['B1'] = 'Sequence'
188. wsExcelNeu['C1'] = 'Seqeunce Length'
189. wsExcelNeu['D1'] = 'Gene'
190. wsExcelNeu['E1'] = 'Peptide Name'
191. wsExcelNeu['F1'] = 'Producer Organism'
192. wsExcelNeu['G1'] = 'Target_Organism'
193. wsExcelNeu['H1'] = 'Activity'
194. wsExcelNeu['I1'] = 'Swiss-/Uni-Prot ID'
195. wsExcelNeu['J1'] = 'PDB ID'
196. wsExcelNeu['K1'] = 'PubMed ID'
197. wsExcelNeu['L1'] = 'Publication Title'
199. wsExcelNeu['A{}'.format(row)] = 'PD_' + str(IDnumber)
200. wsExcelNeu['B{}'.format(row)] = Sequence
201. wsExcelNeu['C{}'.format(row)] = SequenceLength
202. wsExcelNeu['D{}'.format(row)] = Gene
203. wsExcelNeu['E{}'.format(row)] = peptideName
204. wsExcelNeu['F{}'.format(row)] = producerOrganism
205. wsExcelNeu['G{}'.format(row)] = targetOrganism
206. wsExcelNeu['H{}'.format(row)] = Activity
207. wsExcelNeu['I{}'.format(row)] = uniProtID
208. wsExcelNeu['J{}'.format(row)] = PDB
209. wsExcelNeu['K{}'.format(row)] = PubMedID
210. wsExcelNeu['L{}'.format(row)] = publicationTitle
212. wbExelNeu.save(r'repository')

**Source code 2. DBAASP_Database.py**

1. **import** re
2. **import** openpyxl
3. **from** tqdm **import** tqdm
4. **import** time
6. wbExcel = openpyxl.load_workbook('repository')
7. wsExcel = wbExcel['polished PD']
9. organismList = []
10. **for** row **in** tqdm(range(2, 30)):
12. ID = wsExcel.cell(row, 1).value
13. PDOrganisms = wsExcel.cell(row,5).value
15. m = re.findall('[A-Z][a-z]+[ ]{1,}[a-z]+|[A-Z][a-z]+[ ]{1,}[a-z]+[ ]{1,}|[A-Z][.][ ]{1,}[a-z]+|[A-Z][.][ ]{1,}[a-z]+[ ]{1,}', PDOrganisms)
17. **if** m:
18. **for** i **in** m:
19. wordInN = i
20. **if** wordInN **not** **in** organismList:
21. organismList.append(wordInN)
23. **for** i **in** organismList:
24. word = i
25. **if** word **in** PDOrganisms:
27. PDOrganisms = PDOrganisms.replace(word, word + ',')
29. wsExcel['A{}'.format(row)] = ID
30. wsExcel['F{}'.format(row)] = PDOrganisms

33. **print**(organismList)
35. wbExcel.save(r'repository')

**Source code 3. OrganismFindDBAASP.py**

1. **import** re
2. **import** openpyxl
3. **from** tqdm **import** tqdm

6. wbExcel = openpyxl.load_workbook('repository')
7. wsExcel = wbExcel['original (2)']
8. wsExcel2 = wbExcel['target list man edit']
10. organismList = []
11. **for** row **in** tqdm(range(2, 5756)):
13. ID = wsExcel.cell(row, 1).value
14. PDOrganisms = str(wsExcel.cell(row,3).value)
16. m = re.findall('^(.+?),|;(.+?),', PDOrganisms)
17. m = [tuple(j **for** j **in** i **if** j)[-1] **for** i **in** m]
19. **if** m:
20. **for** i **in** m:
21. wordInN = i+', '
22. **if** wordInN **not** **in** organismList:
23. organismList.append(wordInN)
25. **for** i, organism **in** enumerate(organismList):
26. wsExcel2.cell(row=i+1, column=1).value = organism

29. wbExcel.save(r'repository')

**Source code 4. TargetOrganism_1.py**

1. **import** re
2. **import** openpyxl
3. **from** tqdm **import** tqdm

6. wbExcel = openpyxl.load_workbook('repository')
7. wsExcel = wbExcel['original (2)']
8. wsExcel2 = wbExcel['target list man edit']
9. wsExcel3 = wbExcel['target organisms edited']
10. wsExcel4 = wbExcel['target list groups']
12. columnOriginal = wsExcel2['A']
13. columnEdited = wsExcel2['B']
14. columnGroups = wsExcel2['C']

17. **for** row **in** tqdm(range(2, 5755)):
18. ID = wsExcel.cell(row, 1).value
20. TargetsOriginal = [columnOriginal[x].value **for** x **in** range(len(columnOriginal))]
21. TargetsEdited = [columnEdited[x].value **for** x **in** range(len(columnEdited))]
22. dictTargetEditing = dict(zip(TargetsOriginal, TargetsEdited))
24. TargetOrganisms = str(wsExcel.cell(row, 3).value) # old targets
26. m = re.findall('^(.+?),|;(.+?),', TargetOrganisms)
27. m = [tuple(j **for** j **in** i **if** j)[-1] **for** i **in** m]
29. **if** m:
30. **for** i **in** m:
31. **for** TargetsOriginal **in** dictTargetEditing.keys():
32. TargetOrganisms = str(TargetOrganisms.replace(str(TargetsOriginal), str(dictTargetEditing[TargetsOriginal])))
34. TargetOrganisms = TargetOrganisms.replace("None", "")
36. wsExcel3['A{}'.format(row)] = ID
37. wsExcel3['B{}'.format(row)] = TargetOrganisms

40. **for** row **in** tqdm(range(2, 5755)):
41. ID = wsExcel3.cell(row, 1).value
42. TargetOrganisms = str(wsExcel3.cell(row, 2).value)
44. TargetsEdited = [columnEdited[x].value **for** x **in** range(len(columnEdited))]
45. TargetGroups = [columnGroups[x].value **for** x **in** range(len(columnGroups))]
46. dictTargetGroups = dict(zip(TargetsEdited, TargetGroups))
48. m = re.findall('^(.+?),|;(.+?),', TargetOrganisms)
49. m = [tuple(j **for** j **in** i **if** j)[-1] **for** i **in** m]
51. organismList = []
52. FilFungus = 0
53. Yeast = 0
54. GramPos = 0
55. GramNeg = 0
56. Virus = 0
57. MamCell = 0
58. MamCanCell = 0
59. Parasite = 0
60. Other = 0
62. **if** m:
63. **for** i **in** m:
64. wordInN = i+','
65. **if** wordInN **not** **in** organismList:
67. organismList.append(wordInN)
68. FilFungus = 0
69. Yeast = 0
70. GramPos = 0
71. GramNeg = 0
72. Virus = 0
73. MamCell = 0
74. MamCanCell = 0
75. Parasite = 0
76. Other = 0
77. **for** j **in** organismList:
78. **if** 'Fungus' **in** str(dictTargetGroups.get(j)):
79. FilFungus += 1
80. **if** 'Yeast' **in** str(dictTargetGroups.get(j)):
81. Yeast += 1
82. **if** 'Gram+' **in** str(dictTargetGroups.get(j)):
83. GramPos +=1
84. **if** 'Gram-' **in** str(dictTargetGroups.get(j)):
85. GramNeg += 1
86. **if** 'Virus' **in** str(dictTargetGroups.get(j)):
87. Virus += 1
88. **if** 'MamCell' **in** str(dictTargetGroups.get(j)):
89. MamCell += 1
90. **if** 'MamCanCell' **in** str(dictTargetGroups.get(j)):
91. MamCanCell += 1
92. **if** 'Other' **in** str(dictTargetGroups.get(j)):
93. Other += 1
95. **if** FilFungus == 0:
96. FilFungus = ''
97. **if** Yeast == 0:
98. Yeast = ''
99. **if** GramPos == 0:
100. GramPos = ''
101. **if** GramNeg == 0:
102. GramNeg = ''
103. **if** Virus == 0:
104. Virus = ''
105. **if** MamCell == 0:
106. MamCell = ''
107. **if** MamCanCell == 0:
108. MamCanCell = ''
109. **if** Parasite == 0:
110. Parasite = ''
111. **if** Other == 0:
112. Other = ''
114. wsExcel4['A{}'.format(row)] = ID
115. wsExcel4['B{}'.format(row)] = TargetOrganisms
116. wsExcel4['C{}'.format(row)] = ' '
117. wsExcel4['D{}'.format(row)] = FilFungus
118. wsExcel4['E{}'.format(row)] = Yeast
119. wsExcel4['F{}'.format(row)] = GramPos
120. wsExcel4['G{}'.format(row)] = GramNeg
121. wsExcel4['H{}'.format(row)] = Virus
122. wsExcel4['I{}'.format(row)] = MamCell
123. wsExcel4['J{}'.format(row)] = MamCanCell
124. wsExcel4['K{}'.format(row)] = Other

127. wsExcel3['A1'] = 'ID'
128. wsExcel3['B1'] = 'Target Edited'
130. wsExcel4['A1'] = 'ID'
131. wsExcel4['B1'] = 'Target Edited'
132. wsExcel4['C1'] = ' '
133. wsExcel4['D1'] = 'Filamentous Fungus'
134. wsExcel4['E1'] = 'Yeast'
135. wsExcel4['F1'] = 'Gram+'
136. wsExcel4['G1'] = 'Gram-'
137. wsExcel4['H1'] = 'Virus'
138. wsExcel4['I1'] = 'Mammalian cell'
139. wsExcel4['J1'] = 'Mammalian cancer cell'
140. wsExcel4['K1'] = 'Other'
142. wbExcel.save(r'repository')

**Source code 5. TargetOrganism_2.py**

1. **import** openpyxl
2. **from** tqdm **import** tqdm
4. wbExcel = openpyxl.load_workbook(r'repository')
5. wsExcel = wbExcel['Combined']
7. wbExcelPrimaryStructure = openpyxl.load_workbook(r'repository’)
9. **try**:
10. wbExcelPrimaryStructure.remove(wbExcelPrimaryStructure['GRAVY'])
11. **except** KeyError:
12. **pass**
14. wsExcelNeu = wbExcelPrimaryStructure.create_sheet('GRAVY')

17. # Eisenberg et al 1984, "Analysis of Membrane and Surface Protein Sequences with the Hydrophobic Moments Plot", Table 1, Normalized consensus
18. Eisenberg_scale = {'R':-2.53,'K':-1.50,'D':-0.90,'Q':-0.85,'N':-0.78,'E':-0.74,'H':-0.40,'S':-0.18,'T':-0.05,'P':0.12,'Y':0.26,'C':0.29,'G':0.48,'A':0.62,'M':0.64,'W':0.81,'L':1.06,'V':1.08,'F':1.19,'I':1.38}
20. **for** row **in** tqdm(range(2,3830)):
22. ID = wsExcel.cell(row, 1).value
23. Sequence = str(wsExcel.cell(row, 2).value)
24. LengthExcel = str(len(Sequence))
25. gravy_sum = 0
26. count = 0
28. **for** letter **in** Sequence:
29. **try**:
30. gravy_sum += Eisenberg_scale[letter]
31. **except** KeyError:
32. count += count+1
34. gravy_seq = gravy_sum/float(LengthExcel)

37. wsExcelNeu['A1'] = 'ID'
38. wsExcelNeu['B1'] = 'Sequence'
39. wsExcelNeu['C1'] = 'Length'
40. wsExcelNeu['D1'] = ' '
41. wsExcelNeu['E1'] = 'GRAVY'
43. wsExcelNeu['A{}'.format(row)] = ID
44. wsExcelNeu['B{}'.format(row)] = Sequence
45. wsExcelNeu['C{}'.format(row)] = LengthExcel
46. wsExcelNeu['D{}'.format(row)] = ' '
47. wsExcelNeu['E{}'.format(row)] = gravy_seq

50. wbExcelPrimaryStructure.save(r'repository')

**Source code 6. GRAVY.py**

1. **from** selenium **import** webdriver
2. **from** selenium.webdriver.firefox.options **import** Options
3. **from** selenium.webdriver.common.by **import** By
4. **from** selenium.webdriver.support.ui **import** WebDriverWait
5. **from** selenium.webdriver.support **import** expected_conditions as EC
6. **import** openpyxl
7. **from** bs4 **import** BeautifulSoup
8. **from** tqdm **import** tqdm
9. **import** time
11. wbExcel = openpyxl.load_workbook(r'repository')
12. wsExcel = wbExcel['Combined']
14. wbExcelSecondaryStructure = openpyxl.load_workbook(r'repository')
15. wsExcelNeu = wbExcelSecondaryStructure['Gamma Core Motif']
17. options = Options()
18. options.add_argument('--headless')
19. driver = webdriver.Firefox(executable_path=r'D:\Desktop\geckodriver.exe', options=options)
21. count = 0
23. **for** row **in** tqdm(range(2999, 3252)):
25. count += 1
27. driver.get('https://prosite.expasy.org/scanprosite/')
29. # Choose: Submit PROTEIN sequences and MOTIFS to scan them against each other.
30. driver.find_element_by_xpath('/html/body/div[2]/div[2]/div/form/span[3]/input').click()
32. # Enter aa sequence and motif
33. ID = wsExcel.cell(row, 1).value
34. Sequence = wsExcel.cell(row, 2).value
36. **try**:
37. elemValuepI = WebDriverWait(driver, 30).until(
38. EC.presence_of_element_located((By.XPATH, '/html/body/div[2]/div[2]/div/form/div[3]/div[2]/textarea'))
39. )
40. **finally**:
41. elemSeq = driver.find_element_by_xpath('/html/body/div[2]/div[2]/div/form/div[3]/div[1]/div[1]/textarea')
42. elemSeq.clear()
43. elemSeq.send_keys(Sequence)
45. driver.find_element_by_id('meta3_add_opt').click()
47. driver.find_element_by_xpath('/html/body/div[2]/div[2]/div/form/div[3]/div[2]/div/ul/li[2]/ul/li[2]/select/option[5]').click()
49. elemMotif = driver.find_element_by_xpath('/html/body/div[2]/div[2]/div/form/div[3]/div[2]/textarea')
50. elemMotif.clear()
51. elemMotif.send_keys('G-X-C-{C}(3,9)-C')
53. driver.find_element_by_xpath('/html/body/div[2]/div[2]/div/form/div[4]/table/tbody/tr[1]/td[2]/select/option[4]').click()
54. driver.find_element_by_xpath('/html/body/div[2]/div[2]/div/form/div[4]/center/input[1]').click()

57. **try**:
58. elemValuepI = WebDriverWait(driver, 20).until(
59. EC.presence_of_element_located((By.XPATH, '/html/body/div[2]/div[2]/pre'))
60. )
61. **finally**:
62. html = driver.page_source
64. soup = BeautifulSoup(html, features="html.parser")
65. lines = str(soup).split('\n')
67. string=[]
69. dextromeric = ''
70. **for** i **in** lines:
72. **if** 'USERSEQ1' **and** 'USERPAT1' **in** i:
73. **if** 'motif on sequence' **not** **in** i:
74. string.append(i)
76. **for** i **in** string:
78. helper1 = i.split('/')
79. dextromeric += str(helper1[1])+','

82. wsExcelNeu['A{}'.format(row)] = ID
83. wsExcelNeu['B{}'.format(row)] = Sequence
84. wsExcelNeu['C{}'.format(row)] = ' '
85. wsExcelNeu['D{}'.format(row)] = dextromeric
87. **if** count == 500:
88. count = 0
89. wbExcelSecondaryStructure.save(r'repository')
90. time.sleep(30)
92. # to Excel
93. wsExcelNeu['A1'] = 'ID'
94. wsExcelNeu['B1'] = 'Sequence'
95. wsExcelNeu['C1'] = ' '
96. wsExcelNeu['D1'] = 'dextromeric isoform (G-X-C-X(3,9)-C)'
97. wsExcelNeu['E1'] = 'levomeric isoform 1 (C-X(3,9)-C-X-G)'
98. wsExcelNeu['F1'] = 'levomeric isoform 2 (C-X(3,9)-G-X-C)'

101. driver.close()
103. wbExcelSecondaryStructure.save(r'repository')

**Source code 7. GammaCoreMotif_WS_D.py**

1. **from** selenium **import** webdriver
2. **from** selenium.webdriver.firefox.options **import** Options
3. **from** selenium.webdriver.common.by **import** By
4. **from** selenium.webdriver.support.ui **import** WebDriverWait
5. **from** selenium.webdriver.support **import** expected_conditions as EC
6. **import** openpyxl
7. **from** bs4 **import** BeautifulSoup
8. **from** tqdm **import** tqdm
9. **import** time
11. wbExcel = openpyxl.load_workbook(r'repository')
12. wsExcel = wbExcel['Combined']
14. wbExcelSecondaryStructure = openpyxl.load_workbook(r'repository')
15. wsExcelNeu = wbExcelSecondaryStructure['Gamma Core Motif']
17. options = Options()
18. options.add_argument('--headless')
19. driver = webdriver.Firefox(executable_path=r'D:\Desktop\geckodriver.exe', options=options)
21. count = 0
23. **for** row **in** tqdm(range(2, 3252)):
25. count += 1
27. driver.get('https://prosite.expasy.org/scanprosite/')
29. # Choose: Submit PROTEIN sequences and MOTIFS to scan them against each other.
30. driver.find_element_by_xpath('/html/body/div[2]/div[2]/div/form/span[3]/input').click()
32. # Enter aa sequence and motif
33. ID = wsExcel.cell(row, 1).value
34. Sequence = wsExcel.cell(row, 2).value
36. **try**:
37. elemValuepI = WebDriverWait(driver, 30).until(
38. EC.presence_of_element_located((By.XPATH, '/html/body/div[2]/div[2]/div/form/div[3]/div[2]/textarea'))
39. )
40. **finally**:
41. elemSeq = driver.find_element_by_xpath('/html/body/div[2]/div[2]/div/form/div[3]/div[1]/div[1]/textarea')
42. elemSeq.clear()
43. elemSeq.send_keys(Sequence)
45. driver.find_element_by_id('meta3_add_opt').click()
47. driver.find_element_by_xpath('/html/body/div[2]/div[2]/div/form/div[3]/div[2]/div/ul/li[2]/ul/li[2]/select/option[5]').click()
49. elemMotif = driver.find_element_by_xpath('/html/body/div[2]/div[2]/div/form/div[3]/div[2]/textarea')
50. elemMotif.clear()
51. elemMotif.send_keys('C-{C}(3,9)-C-X-G')
53. driver.find_element_by_xpath(
54. '/html/body/div[2]/div[2]/div/form/div[4]/table/tbody/tr[1]/td[2]/select/option[4]').click()
55. driver.find_element_by_xpath('/html/body/div[2]/div[2]/div/form/div[4]/center/input[1]').click()
56. **try**:
57. elemValuepI = WebDriverWait(driver, 20).until(
58. EC.presence_of_element_located((By.XPATH, '/html/body/div[2]/div[2]/pre'))
59. )
60. **finally**:
61. html = driver.page_source
63. soup = BeautifulSoup(html, features="html.parser")
64. lines = str(soup).split('\n')
66. string = []
67. levomeric1 = ''
69. **for** i **in** lines:
71. **if** 'USERSEQ1' **and** 'USERPAT1' **in** i:
72. **if** 'motif on sequence' **not** **in** i:
73. string.append(i)
75. **for** i **in** string:
77. helper1 = i.split('/')
78. levomeric1 += str(helper1[1]) + ','
80. wsExcelNeu['A{}'.format(row)] = ID
81. wsExcelNeu['B{}'.format(row)] = Sequence
82. wsExcelNeu['C{}'.format(row)] = ' '
83. wsExcelNeu['E{}'.format(row)] = levomeric1
85. **if** count == 500:
86. count = 0
87. wbExcelSecondaryStructure.save(r'repository')
88. time.sleep(30)
90. driver.close()
92. wbExcelSecondaryStructure.save(r'repository')

**Source code 8. GammaCoreMotif_WS_L1.py**

1. **from** selenium **import** webdriver
2. **from** selenium.webdriver.firefox.options **import** Options
3. **from** selenium.webdriver.common.by **import** By
4. **from** selenium.webdriver.support.ui **import** WebDriverWait
5. **from** selenium.webdriver.support **import** expected_conditions as EC
6. **import** openpyxl
7. **from** bs4 **import** BeautifulSoup
8. **from** tqdm **import** tqdm
9. **import** time
11. wbExcel = openpyxl.load_workbook(r'repository')
12. wsExcel = wbExcel['Combined']
14. wbExcelSecondaryStructure = openpyxl.load_workbook(r'repository')
15. wsExcelNeu = wbExcelSecondaryStructure['Gamma Core Motif']
17. options = Options()
18. options.add_argument('--headless')
19. driver = webdriver.Firefox(executable_path=r'D:\Desktop\geckodriver.exe', options=options)
21. count = 0
23. **for** row **in** tqdm(range(2, 3252)):
25. count += 1
27. driver.get('https://prosite.expasy.org/scanprosite/')
29. # Choose: Submit PROTEIN sequences and MOTIFS to scan them against each other.
30. driver.find_element_by_xpath('/html/body/div[2]/div[2]/div/form/span[3]/input').click()
32. # Enter aa sequence and motif
33. ID = wsExcel.cell(row, 1).value
34. Sequence = wsExcel.cell(row, 2).value
36. **try**:
37. elemValuepI = WebDriverWait(driver, 30).until(
38. EC.presence_of_element_located((By.XPATH, '/html/body/div[2]/div[2]/div/form/div[3]/div[2]/textarea'))
39. )
40. **finally**:
41. elemSeq = driver.find_element_by_xpath('/html/body/div[2]/div[2]/div/form/div[3]/div[1]/div[1]/textarea')
42. elemSeq.clear()
43. elemSeq.send_keys(Sequence)
45. driver.find_element_by_id('meta3_add_opt').click()
47. driver.find_element_by_xpath('/html/body/div[2]/div[2]/div/form/div[3]/div[2]/div/ul/li[2]/ul/li[2]/select/option[5]').click()
49. elemMotif = driver.find_element_by_xpath('/html/body/div[2]/div[2]/div/form/div[3]/div[2]/textarea')
50. elemMotif.clear()
51. elemMotif.send_keys('C-{C}(3,9)-G-X-C')
53. driver.find_element_by_xpath(
54. '/html/body/div[2]/div[2]/div/form/div[4]/table/tbody/tr[1]/td[2]/select/option[4]').click()
55. driver.find_element_by_xpath('/html/body/div[2]/div[2]/div/form/div[4]/center/input[1]').click()
56. **try**:
57. elemValuepI = WebDriverWait(driver, 20).until(
58. EC.presence_of_element_located((By.XPATH, '/html/body/div[2]/div[2]/pre'))
59. )
60. **finally**:
61. html = driver.page_source
63. soup = BeautifulSoup(html, features="html.parser")
64. lines = str(soup).split('\n')
66. string = []
67. levomeric2 = ''
69. **for** i **in** lines:
70. **if** 'USERSEQ1' **and** 'USERPAT1' **in** i:
71. **if** 'motif on sequence' **not** **in** i:
72. string.append(i)
74. **for** i **in** string:
75. helper1 = i.split('/')
76. levomeric2 += str(helper1[1]) + ','
78. wsExcelNeu['A{}'.format(row)] = ID
79. wsExcelNeu['B{}'.format(row)] = Sequence
80. wsExcelNeu['C{}'.format(row)] = ' '
81. wsExcelNeu['F{}'.format(row)] = levomeric2
83. **if** count == 500:
84. count = 0
85. wbExcelSecondaryStructure.save(r'repository')
86. time.sleep(30)
88. driver.close()
90. wbExcelSecondaryStructure.save(r'repository')

**Source code 9. GammaCoreMotif_WS_L2.py**

1. **from** selenium **import** webdriver
2. **from** selenium.webdriver.common.keys **import** Keys
3. **from** selenium.webdriver.firefox.options **import** Options
4. **from** selenium.webdriver.common.by **import** By
5. **from** selenium.webdriver.support.ui **import** WebDriverWait
6. **from** selenium.webdriver.support **import** expected_conditions as EC
7. **import** openpyxl
8. **from** bs4 **import** BeautifulSoup
9. **from** tqdm **import** tqdm
11. wbExcelSecondaryStructure = openpyxl.load_workbook(r'repository')
12. wsExcelNeu = wbExcelSecondaryStructure['Gamma Core Motif']

15. options = Options()
16. options.add_argument('--headless')
17. driver = webdriver.Firefox(executable_path=r'D:\Desktop\geckodriver.exe', options=options)
19. # motif seqeunce(Yount 2004)
20. # pos net charge pH 7.4(ProteinCalculator?)
21. # hydrophobic(negativ GRAVY)

24. # Eisenberg et al 1984, "Analysis of Membrane and Surface Protein Sequences with the Hydrophobic Moments Plot", Table 1, Normalized consensus
25. Eisenberg_scale = {'R':-2.53,'K':-1.50,'D':-0.90,'Q':-0.85,'N':-0.78,'E':-0.74,'H':-0.40,'S':-0.18,'T':-0.05,'P':0.12,'Y':0.26,'C':0.29,'G':0.48,'A':0.62,'M':0.64,'W':0.81,'L':1.06,'V':1.08,'F':1.19,'I':1.38}
27. **for** row **in** tqdm(range(2, 3252)):
29. ID = wsExcelNeu.cell(row, 1).value
30. Sequence = wsExcelNeu.cell(row, 2).value
31. LengthExcel = str(len(Sequence))
33. GammaCoreD = wsExcelNeu.cell(row, 4).value
34. GammaCoreL1 = wsExcelNeu.cell(row, 5).value
35. GammaCoreL2 = wsExcelNeu.cell(row, 6).value
36. verifiedMotif = ''
38. **try**:
39. GammaCoreDRange = GammaCoreD.split(',')[:-1]
41. **for** i **in** GammaCoreDRange:
43. motifStart = int(i.split('-')[0])-1
44. motifStop = int(i.split('-')[1])
45. motifSequence = Sequence[motifStart:motifStop]


49. # -------------------- What is the charge of the motif ? --------------------#
50. driver.get("http://protcalc.sourceforge.net/")
52. elem = driver.find_element_by_name("seq")
53. elem.clear()
54. elem.send_keys(motifSequence)
56. # Choose charge at pH 7.0
57. driver.find_element_by_xpath("/html/body/form/center[6]/table/tbody/tr[1]/td[2]/input[2]").click()
58. elemCharge74 = driver.find_element_by_xpath("/html/body/form/center[6]/table/tbody/tr[1]/td[2]/input[3]")
59. driver.execute_script("arguments[0].value = '7.4';", elemCharge74)
61. # Submit Form
62. elemSubmit = driver.find_element_by_xpath("/html/body/form/center[7]/input")
63. elemSubmit.send_keys(Keys.ENTER)
64. ########################################################################################################################
65. # Data scrubing and give in Excel file
67. **try**:
68. elemValuepI = WebDriverWait(driver, 30).until(
69. EC.presence_of_element_located((By.XPATH, "/html/body/table/tbody/tr/td[1]/a"))
70. )
71. **except**:
72. **pass**
73. **finally**:
74. html = driver.page_source

77. soup = BeautifulSoup(html, features="html.parser")
78. lines = str(soup).split('\n')
80. # get Charge at pH 7.4
81. **for** i **in** lines:
82. **if** "charge at pH 7.40" **in** i:
84. ChargeAtPh74 = str(i)[42:-14]
86. # -------------------- Is the charge of the motif positive ? --------------------#
88. **if** float(ChargeAtPh74) > 0:
89. # -------------------- Is the GRAVY of the  motif negative = hydrophobic? --------------------#
90. gravy_sum = 0
91. count = 0
93. **for** letter **in** motifSequence:
94. **try**:
95. gravy_sum += Eisenberg_scale[letter]
96. **except** KeyError:
97. count += count + 1
99. Length = float(LengthExcel) - count
100. gravy_seq = gravy_sum / Length
102. **if** gravy_seq < 0:
103. verifiedMotif += str(motifStart+1)+'-'+str(motifStop)+','
105. **else**:
106. wsExcelNeu['H{}'.format(row)] = ''
107. **else**:
108. wsExcelNeu['H{}'.format(row)] = ''
110. wsExcelNeu['H{}'.format(row)] = verifiedMotif
112. **except** AttributeError:
113. wsExcelNeu['H{}'.format(row)] = ''
114. **pass**
116. verifiedMotif = ''
118. **try**:
119. GammaCoreL1Range = GammaCoreL1.split(',')[:-1]
121. **for** i **in** GammaCoreL1Range:
123. motifStart = int(i.split('-')[0])-1
124. motifStop = int(i.split('-')[1])
125. motifSequence = Sequence[motifStart:motifStop]


129. # -------------------- What is the charge of the motif ? --------------------#
131. driver.get("http://protcalc.sourceforge.net/")
133. elem = driver.find_element_by_name("seq")
134. elem.clear()
135. elem.send_keys(motifSequence)
137. # Choose charge at pH 7.0
138. driver.find_element_by_xpath("/html/body/form/center[6]/table/tbody/tr[1]/td[2]/input[2]").click()
139. elemCharge74 = driver.find_element_by_xpath("/html/body/form/center[6]/table/tbody/tr[1]/td[2]/input[3]")
140. driver.execute_script("arguments[0].value = '7.4';", elemCharge74)
142. # Submit Form
143. elemSubmit = driver.find_element_by_xpath("/html/body/form/center[7]/input")
144. elemSubmit.send_keys(Keys.ENTER)
145. ########################################################################################################################
146. # Data scrubing and give in Excel file
148. **try**:
149. elemValuepI = WebDriverWait(driver, 30).until(
150. EC.presence_of_element_located((By.XPATH, "/html/body/table/tbody/tr/td[1]/a"))
151. )
152. **except**:
153. **pass**
154. **finally**:
155. html = driver.page_source

158. soup = BeautifulSoup(html, features="html.parser")
159. lines = str(soup).split('\n')
161. # get Charge at pH 7.4
162. **for** i **in** lines:
163. **if** "charge at pH 7.40" **in** i:
165. ChargeAtPh74 = str(i)[42:-14]
167. # -------------------- Is the charge of the motif positive ? --------------------#
169. **if** float(ChargeAtPh74) > 0:
170. # -------------------- Is the GRAVY of the  motif negative = hydrophobic? --------------------#
171. gravy_sum = 0
172. count = 0
174. **for** letter **in** motifSequence:
175. **try**:
176. gravy_sum += Eisenberg_scale[letter]
177. **except** KeyError:
178. count += count + 1
180. Length = float(LengthExcel) - count
181. gravy_seq = gravy_sum / Length
183. **if** gravy_seq < 0:
184. verifiedMotif += str(motifStart+1)+'-'+str(motifStop)+','
186. **else**:
187. wsExcelNeu['I{}'.format(row)] = ''
188. **else**:
189. wsExcelNeu['I{}'.format(row)] = ''
191. wsExcelNeu['I{}'.format(row)] = verifiedMotif
193. **except** AttributeError:
194. wsExcelNeu['I{}'.format(row)] = ''
195. **pass**
197. verifiedMotif = ''
199. **try**:
200. GammaCoreL2Range = GammaCoreL2.split(',')[:-1]
202. **for** i **in** GammaCoreL2Range:
204. motifStart = int(i.split('-')[0])-1
205. motifStop = int(i.split('-')[1])
206. motifSequence = Sequence[motifStart:motifStop]


210. # -------------------- What is the charge of the motif ? --------------------#
212. driver.get("http://protcalc.sourceforge.net/")
214. elem = driver.find_element_by_name("seq")
215. elem.clear()
216. elem.send_keys(motifSequence)
218. # Choose charge at pH 7.0
219. driver.find_element_by_xpath("/html/body/form/center[6]/table/tbody/tr[1]/td[2]/input[2]").click()
220. elemCharge74 = driver.find_element_by_xpath("/html/body/form/center[6]/table/tbody/tr[1]/td[2]/input[3]")
221. driver.execute_script("arguments[0].value = '7.4';", elemCharge74)
223. # Submit Form
224. elemSubmit = driver.find_element_by_xpath("/html/body/form/center[7]/input")
225. elemSubmit.send_keys(Keys.ENTER)
226. ########################################################################################################################
227. # Data scrubing and give in Excel file
229. **try**:
230. elemValuepI = WebDriverWait(driver, 30).until(
231. EC.presence_of_element_located((By.XPATH, "/html/body/table/tbody/tr/td[1]/a"))
232. )
233. **except**:
234. **pass**
235. **finally**:
236. html = driver.page_source

239. soup = BeautifulSoup(html, features="html.parser")
240. lines = str(soup).split('\n')
242. # get Charge at pH 7.4
243. **for** i **in** lines:
244. **if** "charge at pH 7.40" **in** i:
246. ChargeAtPh74 = str(i)[42:-14]
248. # -------------------- Is the charge of the motif positive ? --------------------#
250. **if** float(ChargeAtPh74) > 0:
251. # -------------------- Is the GRAVY of the  motif negative = hydrophobic? --------------------#
252. gravy_sum = 0
253. count = 0
255. **for** letter **in** motifSequence:
256. **try**:
257. gravy_sum += Eisenberg_scale[letter]
258. **except** KeyError:
259. count += count + 1
261. Length = float(LengthExcel) - count
262. gravy_seq = gravy_sum / Length
264. **if** gravy_seq < 0:
265. verifiedMotif += str(motifStart+1)+'-'+str(motifStop)+','
267. **else**:
268. wsExcelNeu['J{}'.format(row)] = ''
269. **else**:
270. wsExcelNeu['J{}'.format(row)] = ''
272. wsExcelNeu['J{}'.format(row)] = verifiedMotif
274. **except** AttributeError:
275. wsExcelNeu['J{}'.format(row)] = ''
276. **pass**
278. wbExcelSecondaryStructure.save(r'repository')

**Source code 10. GammaCoreMotifValidation.py**

1. **from** py._builtin **import** execfile
2. **from** selenium **import** webdriver
3. **from** selenium.webdriver.common.keys **import** Keys
4. **from** selenium.webdriver.firefox.options **import** Options
5. **from** selenium.webdriver.common.by **import** By
6. **from** selenium.webdriver.support.ui **import** WebDriverWait
7. **from** selenium.webdriver.support **import** expected_conditions as EC
8. **import** openpyxl
9. **from** bs4 **import** BeautifulSoup
10. **from** tqdm **import** tqdm
11. **import** time


15. wbExcel = openpyxl.load_workbook(r'repository')
16. wsExcel = wbExcel['Combined']
18. wbExcelPrimaryStructure = openpyxl.load_workbook(r'repository')
19. wsExcelNeu = wbExcelPrimaryStructure['pI + mol.weight + charge pH 7.4']
20. wsExcelNeu2 = wbExcelPrimaryStructure["AA Residues"]
22. options = Options()
23. options.add_argument('--headless')
24. driver = webdriver.Firefox(executable_path=r'D:\Desktop\geckodriver.exe', options=options)
26. count = 0
28. **for** row **in** tqdm(range(2, 3252)):
29. count += 1
30. driver.get("http://protcalc.sourceforge.net/")
31. # Enter aa sequence
32. ID = wsExcel.cell(row, 1).value
33. Sequence = wsExcel.cell(row, 2).value
34. Length = len(Sequence)
35. elem = driver.find_element_by_name("seq")
36. elem.clear()
37. elem.send_keys(Sequence)
39. # Choose Molecular weight
40. driver.find_element_by_xpath("/html/body/form/center[6]/table/tbody/tr[1]/td[1]/input[1]").click()
42. # Choose Isoelectric point
43. driver.find_element_by_xpath("/html/body/form/center[6]/table/tbody/tr[1]/td[2]/input[1]").click()
45. # Choose charge at pH 7.4
46. driver.find_element_by_xpath("/html/body/form/center[6]/table/tbody/tr[1]/td[2]/input[2]").click()
47. elemCharge74 = driver.find_element_by_xpath("/html/body/form/center[6]/table/tbody/tr[1]/td[2]/input[3]")
48. driver.execute_script("arguments[0].value = '7.4';", elemCharge74)
50. # Choose Count Residues
51. driver.find_element_by_xpath("/html/body/form/center[6]/table/tbody/tr[2]/td[2]/input[1]").click()
53. # Submit Form
54. elemSubmit = driver.find_element_by_xpath("/html/body/form/center[7]/input")
55. elemSubmit.send_keys(Keys.ENTER)
56. ########################################################################################################################
57. # Data scrubing and give in Excel file
59. **try**:
60. elemValuepI = WebDriverWait(driver, 30).until(
61. EC.presence_of_element_located((By.XPATH, "/html/body/center[6]/h2"))
62. )
63. **finally**:
64. html = driver.page_source
66. soup = BeautifulSoup(html, features="html.parser")
67. lines = str(soup).split('\n')
69. # get pI
70. **for** i **in** lines:
71. **if** "Estimated pI" **in** i:
72. ValuepI = str(i)[27:-19]
74. # get Molecular weight
75. **for** i **in** lines:
76. **if** "Molecular Weight" **in** i:
77. MolecularWeight = str(i)[53:-24]
79. # get Charge at pH 7.4
80. **for** i **in** lines:
81. **if** "charge at pH 7.40" **in** i:
82. ChargeAtPh74 = str(i)[42:-14]
84. # get aa residues
85. control = False
86. **for** i **in** lines:
87. **if** control:
88. Alanine = str(i)[21:-5]
89. **if** "Alanine" **in** i:
90. control  =True
91. **else**:
92. control = False
94. control = False
95. **for** i **in** lines:
96. **if** control:
97. Arginine = str(i)[21:-5]
98. **if** "Arginine" **in** i:
99. control  =True
100. **else**:
101. control = False
103. control = False
104. **for** i **in** lines:
105. **if** control:
106. Asparagine = str(i)[21:-5]
107. **if** "Asparagine" **in** i:
108. control  =True
109. **else**:
110. control = False
112. control = False
113. **for** i **in** lines:
114. **if** control:
115. Aspartate = str(i)[21:-5]
116. **if** "Aspartate" **in** i:
117. control  =True
118. **else**:
119. control = False
121. control = False
122. **for** i **in** lines:
123. **if** control:
124. Glutamine = str(i)[21:-5]
125. **if** "Glutamine" **in** i:
126. control  =True
127. **else**:
128. control = False
130. control = False
131. **for** i **in** lines:
132. **if** control:
133. Glutamate = str(i)[21:-5]
134. **if** "Glutamate" **in** i:
135. control  =True
136. **else**:
137. control = False
139. control = False
140. **for** i **in** lines:
141. **if** control:
142. Glycine = str(i)[21:-5]
143. **if** "Glycine" **in** i:
144. control  =True
145. **else**:
146. control = False
148. control = False
149. **for** i **in** lines:
150. **if** control:
151. Histidine = str(i)[21:-5]
152. **if** "Histidine" **in** i:
153. control  =True
154. **else**:
155. control = False
157. control = False
158. **for** i **in** lines:
159. **if** control:
160. Isoleucine = str(i)[21:-5]
161. **if** "Isoleucine" **in** i:
162. control  =True
163. **else**:
164. control = False
166. control = False
167. **for** i **in** lines:
168. **if** control:
169. Leucine = str(i)[21:-5]
170. **if** "Leucine" **in** i:
171. control  =True
172. **else**:
173. control = False
175. control = False
176. **for** i **in** lines:
177. **if** control:
178. Lysine = str(i)[21:-5]
179. **if** "Lysine" **in** i:
180. control = True
181. **else**:
182. control = False
184. control = False
185. **for** i **in** lines:
186. **if** control:
187. Methionine = str(i)[21:-5]
188. **if** "Methionine" **in** i:
189. control = True
190. **else**:
191. control = False
193. control = False
194. **for** i **in** lines:
195. **if** control:
196. Phenylalanine = str(i)[21:-5]
197. **if** "Phenylalanine" **in** i:
198. control = True
199. **else**:
200. control = False
202. control = False
203. **for** i **in** lines:
204. **if** control:
205. Proline = str(i)[21:-5]
206. **if** "Proline" **in** i:
207. control = True
208. **else**:
209. control = False
211. control = False
212. **for** i **in** lines:
213. **if** control:
214. Serine = str(i)[21:-5]
215. **if** "Serine" **in** i:
216. control = True
217. **else**:
218. control = False
220. control = False
221. **for** i **in** lines:
222. **if** control:
223. Threonine = str(i)[21:-5]
224. **if** "Threonine" **in** i:
225. control = True
226. **else**:
227. control = False
229. control = False
230. **for** i **in** lines:
231. **if** control:
232. Tyrosine = str(i)[21:-5]
233. **if** "Tyrosine" **in** i:
234. control = True
235. **else**:
236. control = False
238. control = False
239. **for** i **in** lines:
240. **if** control:
241. Valine = str(i)[21:-5]
242. **if** "Valine" **in** i:
243. control = True
244. **else**:
245. control = False
247. control = False
248. **for** i **in** lines:
249. **if** control:
250. Tryptophan = str(i)[21:-5]
251. **if** "Tryptophan" **in** i:
252. control = True
253. **else**:
254. control = False
256. control = False
257. **for** i **in** lines:
258. **if** control:
259. Cysteine = str(i)[21:-5]
260. **if** "Cysteine" **in** i:
261. control = True
262. **else**:
263. control = False
265. control = False
266. **for** i **in** lines:
267. **if** control:
268. Selenocysteine = str(i)[21:-5]
269. **if** "Selenocysteine" **in** i:
270. control = True
271. **else**:
272. control = False
274. # to Excel
275. wsExcelNeu2['A{}'.format(row)] = ID
276. wsExcelNeu2['B{}'.format(row)] = Sequence
277. wsExcelNeu2['C{}'.format(row)] = Length
278. wsExcelNeu2['D{}'.format(row)] = ' '
279. wsExcelNeu2['E{}'.format(row)] = int(Alanine)
280. wsExcelNeu2['F{}'.format(row)] = int(Arginine)
281. wsExcelNeu2['G{}'.format(row)] = int(Asparagine)
282. wsExcelNeu2['H{}'.format(row)] = int(Aspartate)
283. wsExcelNeu2['I{}'.format(row)] = int(Glutamine)
284. wsExcelNeu2['J{}'.format(row)] = int(Glutamate)
285. wsExcelNeu2['K{}'.format(row)] = int(Glycine)
286. wsExcelNeu2['L{}'.format(row)] = int(Histidine)
287. wsExcelNeu2['M{}'.format(row)] = int(Isoleucine)
288. wsExcelNeu2['N{}'.format(row)] = int(Leucine)
289. wsExcelNeu2['O{}'.format(row)] = int(Lysine)
290. wsExcelNeu2['P{}'.format(row)] = int(Methionine)
291. wsExcelNeu2['Q{}'.format(row)] = int(Phenylalanine)
292. wsExcelNeu2['R{}'.format(row)] = int(Proline)
293. wsExcelNeu2['S{}'.format(row)] = int(Serine)
294. wsExcelNeu2['T{}'.format(row)] = int(Threonine)
295. wsExcelNeu2['U{}'.format(row)] = int(Tyrosine)
296. wsExcelNeu2['V{}'.format(row)] = int(Valine)
297. wsExcelNeu2['W{}'.format(row)] = int(Tryptophan)
298. wsExcelNeu2['X{}'.format(row)] = int(Cysteine)
299. wsExcelNeu2['Y{}'.format(row)] = int(Selenocysteine)
301. wsExcelNeu['A{}'.format(row)] = ID
302. wsExcelNeu['B{}'.format(row)] = Sequence
303. wsExcelNeu['C{}'.format(row)] = ' '
304. wsExcelNeu['D{}'.format(row)] = float(ValuepI)
305. wsExcelNeu['E{}'.format(row)] = float(MolecularWeight)
306. wsExcelNeu['F{}'.format(row)] = float(ChargeAtPh74)
308. **if** count == 500:
309. count = 0
310. wbExcelPrimaryStructure.save(r'repository')
311. time.sleep(300)
313. wsExcelNeu['A1'] = 'ID'
314. wsExcelNeu['B1'] = 'Sequence'
315. wsExcelNeu['C1'] = ' '
316. wsExcelNeu['D1'] = 'pI'
317. wsExcelNeu['E1'] = 'Molecular Weight'
318. wsExcelNeu['F1'] = 'charge pH 7.4'
320. wsExcelNeu2['A1'] = 'ID'
321. wsExcelNeu2['B1'] = 'Sequence'
322. wsExcelNeu2['C1'] = 'Length'
323. wsExcelNeu2['D1'] = ' '
324. wsExcelNeu2['E1'] = 'Alanine (A)'
325. wsExcelNeu2['F1'] = 'Arginine (R)'
326. wsExcelNeu2['G1'] = 'Asparagine (N)'
327. wsExcelNeu2['H1'] = 'Aspartate (D)'
328. wsExcelNeu2['I1'] = 'Glutamine (Q)'
329. wsExcelNeu2['J1'] = 'Glutamate (E)'
330. wsExcelNeu2['K1'] = 'Glycine (G)'
331. wsExcelNeu2['L1'] = 'Histidine (H)'
332. wsExcelNeu2['M1'] = 'Isoleucine (I)'
333. wsExcelNeu2['N1'] = 'Leucine (L)'
334. wsExcelNeu2['O1'] = 'Lysine (K)'
335. wsExcelNeu2['P1'] = 'Methionine (M)'
336. wsExcelNeu2['Q1'] = 'Phenylalanine (F)'
337. wsExcelNeu2['R1'] = 'Proline (P)'
338. wsExcelNeu2['S1'] = 'Serine (S)'
339. wsExcelNeu2['T1'] = 'Threonine (T)'
340. wsExcelNeu2['U1'] = 'Tyrosine (Y)'
341. wsExcelNeu2['V1'] = 'Valine (V)'
342. wsExcelNeu2['W1'] = 'Tryptophan (W)'
343. wsExcelNeu2['X1'] = 'Cysteine (C)'
344. wsExcelNeu2['Y1'] = 'Selenocysteine (U)'
346. driver.close()
348. wbExcelPrimaryStructure.save(r'repository')

**Source code 11. ProteinCalculator.py**
